# Prolactin receptor-driven combined luminal and epithelial differentiation in breast cancer restricts plasticity, stemness, tumorigenesis and metastasis

**DOI:** 10.1038/s41389-020-00297-5

**Published:** 2021-01-14

**Authors:** Anwar Shams, Najat Binothman, Julien Boudreault, Ni Wang, Fuad Shams, Dana Hamam, Jun Tian, Alaa Moamer, Meiou Dai, Jean-Jacques Lebrun, Suhad Ali

**Affiliations:** 1 Department of Medicine, Cancer Research Program, The Research Institute of the McGill University Health Centre, Montreal, QC Canada; 2 Department of Pathology and Laboratory Medicine, King Abdulaziz Hospital, Mecca, Saudi Arabia; 3 Present Address: Department of Pharmacology, Faculty of Medicine, Taif University, Taif, Saudi Arabia; 4 Present Address: Department of Chemistry, College of Science and Arts, King Abdulaziz University, P.O. Box 344, Rabigh, 21911 Saudi Arabia

**Keywords:** Breast cancer, Cancer stem cells

## Abstract

Dedifferentiation increased cellular plasticity and stemness are established derivers of tumor heterogeneity, metastasis and therapeutic failure resulting in incurable cancers. Therefore, it is essential to decipher pro/forward-differentiation mechanisms in cancer that may serve as therapeutic targets. We found that interfering with expression of the receptor for the lactogenic hormone prolactin (PRLR) in breast cancer cells representative of the luminal and epithelial breast cancer subtypes (hormone receptor positive (HR+) and HER2-enriched (HER2-E) resulted in loss of their differentiation state, enriched for stem-like cell subpopulations, and increased their tumorigenic capacity in a subtype-specific manner. Loss of PRLR expression in HR+ breast cancer cells caused their dedifferentiation generating a mesenchymal-basal-like phenotype enriched in CD44+ breast cancer stem-like cells (BCSCs) showing high tumorigenic and metastatic capacities and resistance to anti-hormonal therapy. Whereas loss of PRLR expression in HER2-E breast cancer cells resulted in loss of their luminal differentiation yet enriched for epithelial ALDH+ BCSC population showing elevated HER2-driven tumorigenic, multi-organ metastatic spread, and resistance to anti-HER2 therapy. Collectively, this study defines PRLR as a driver of precise luminal and epithelial differentiation limiting cellular plasticity, stemness, and tumorigenesis and emphasizing the function of pro/forward-differentiation pathways as a foundation for the discovery of anti-cancer therapeutic targets.

## Introduction

Breast cancer is the most common cancer in women and morbidity and mortality from breast cancer are expected to continue to rise globally^1,2^. A vast majority (over 80%) of breast cancer-related deaths are due to tumor relapse and progression to a metastatic disease for which there is no current effective treatment modality^3,4^. In solid epithelial tumors the significance of tumor differentiation state has long been known as a fundamental clinical marker in predicting tumor behavior. Indeed, in breast cancer, metastasis and recurrence have been proposed to be a consequence of loss of tumor cellular differentiation and increased plasticity (dedifferentiation) with loss of both luminal and epithelial features conversely gain of mesenchymal/migratory and stem-like features^5–7^. Because of this important link between tumor differentiation state and cancer severity, stemness and poor outcome, targeting tumor cell plasticity through differentiation induction therapy has been proposed as a potential viable approach to reverse and suppress cancer aggressive phenotype^8–10^. Indeed, differentiation therapy has already shown success in treating hematological malignancies and its application in solid tumor models, such as breast cancer, is gaining momentum^11,12^.

In line with the complex nature of mammary gland development and differentiation, extensive in vitro and in vivo studies have demonstrated the indispensable role of the lactogenic hormone prolactin (PRL) and downstream signaling pathway (PRLR/Jak2/Stat5a/b) in promoting multiple aspects of mammary differentiation program and lactation^13–15^. Our understanding of the role of PRL in breast cancer remains however incomplete. Several studies have described PRL as an autocrine/paracrine factor in mammary epithelial cells promoting cell viability and tumorigenesis^16–18^. However, translation of these findings to therapeutic modalities in breast cancer were not promising^19,20^. In fact, other studies have challenged the autocrine role of PRL as a pro-oncogenic factor^21–25^. Also, restoring and activating the PRL pathway was found to supress breast cancer stem-like cell (BCSC) subpopulations CD44+/CD24− and ALDH+ and reduced breast tumorigenesis in vitro and in vivo^23,26,27^. Moreover, studies examining large cohorts of human breast cancer cases defined PRLR and PRL as markers of favorable clinicopathological parameters (tumor differentiation) and better patient survival outcomes^28–31^. Together, these findings highlight the complex nature of PRL role in breast cancer.

Current breast cancer classification defines the luminal hormone receptor positive (HR+), HER2-enriched (HER2-E) (HR−/HER2 overexpressing), and the triple negative (TNBC) (ER−/PR−/HER2−) groups, as the main basic subtypes^32,33^. Here, using CRISPR/Cas9 technology, we interfered with expression of the PRLR in two different breast cancer cell model systems representative of the two luminal/epithelial breast cancer subtypes HR+ (MCF-7 cells) and HER2-E (SKBR-3 cells). Interestingly, our results showed that suppression of PRLR expression promoted tumor cellular plasticity and tumorigenicity in both breast cancer subtypes. Indeed, our findings demonstrate that PRLR expression in breast cancer is indispensable in deriving both luminal and epithelial differentiation necessary to suppress stemness and diverse features of cancer aggressiveness and highlight PRLR as a pro/forward-differentiation therapeutic target in breast cancer.

## Results

### Loss of PRLR expression in HR+ and HER2-E human breast cancer cells promotes cellular viability, migration, and invasion capacities

**Fig. 1:**
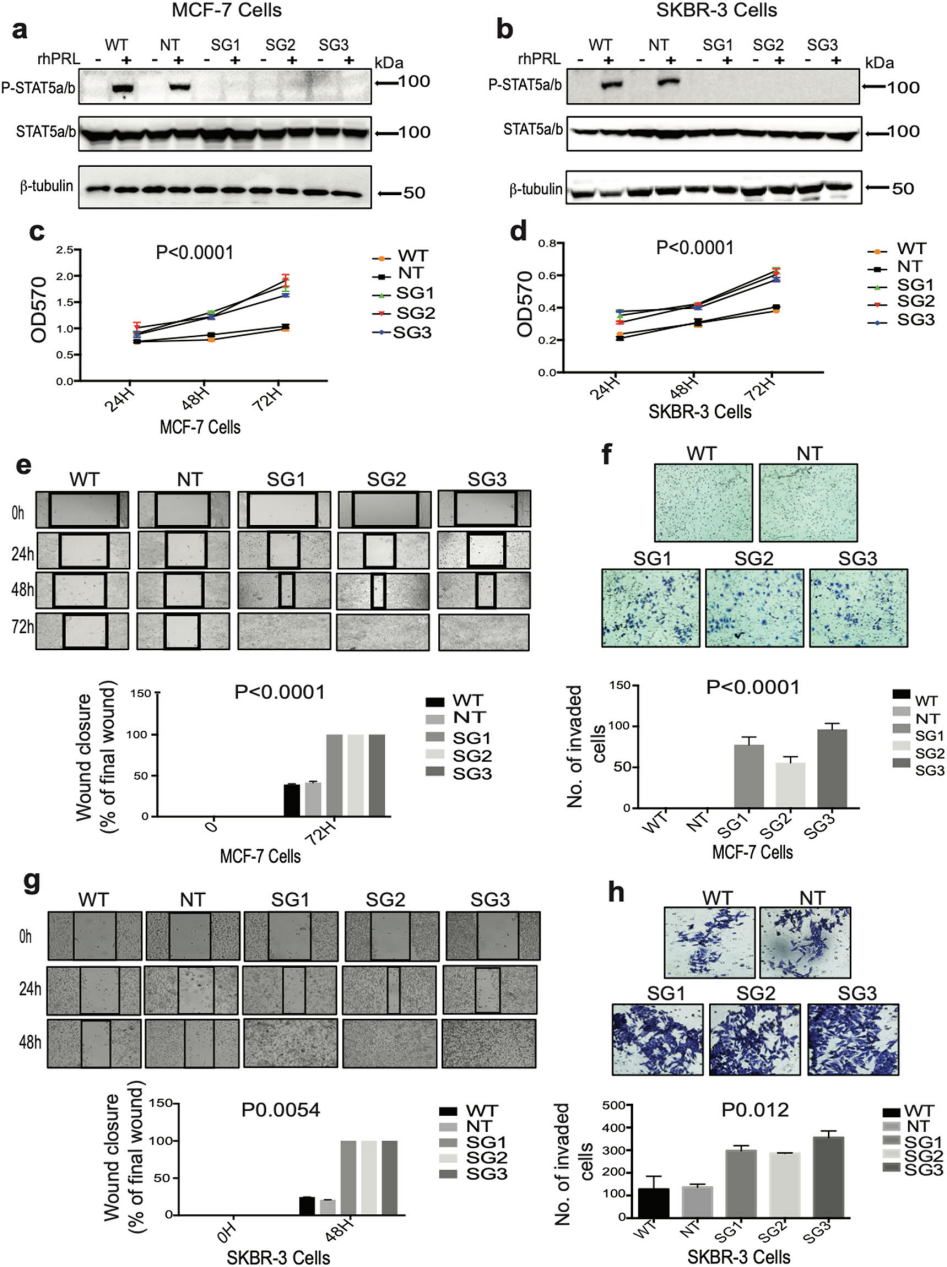
Loss of PRLR expression in breast cancer cells blocked PRL signaling and enhanced cell viability, migration, and invasion potential. **a**, **b** Immunoblot analyses of total cell lysates of MCF-7/WT, MCF-7/NT, and MCF-7/PRLRKO (SG1, SG2, and SG3) as well as SKBR-3/WT, SKBR-3/NT, and SKBR-3/PRLRKO (SG1, SG2, and SG3) following stimulation with rhPRL (250 ng/ml) for 15 min using antibodies against Phospho-STAT5a/b, STAT5a/b, and β-tubulin. **c**, **d** MTT assays were performed using MCF-7/WT, MCF-7/NT, and MCF-7/PRLRKO (SG1, SG2, and SG3) cell lines as well as SKBR-3/WT, SKBR-3/NT, and SKBR-3/PRLRKO (SG1, SG2, and SG3) cell lines for 24, 48, and 72 h. Results are expressed as mean ± SEM of four replicates of three independent experiments ****P < 0.0001 (two-way ANOVA). **e** MCF-7/WT, MCF-7/NT, and MCF-7/PRLRKO (SG1, SG2, and SG3) cell lines were subjected to scratch wound assay. Cells were analyzed at 24, 48, and 72 h. Upper panels show representative cell migration experiment. Lower panels quantification of results of four replicates of three independent experiments expressed as mean ± SEM ****P< 0.0001 (two-way ANOVA). **f** Quantitative invasion assays of MCF-7/WT, MCF-7/NT, and MCF-7/PRLRKO (SG1, SG2, and SG3) cell lines. Upper panels show representative cell invasion experiment. Lower panel, quantification of results of four replicates of four independent experiments ****P < 0.0001 (one-way ANOVA). **g** SKBR-3/WT, SKBR-3/NT, and SKBR-3/PRLRKO (SG1, SG2, and SG3) cell lines were subjected to scratch wound assay. Cells were analyzed at 24 and 48 h. Upper panels show representative cell migration experiment. Lower panels show quantification of results of four replicates of three independent experiments expressed as mean ± SEM of ***P 0.00054 (two-way ANOVA). **h** Quantitative invasion assays of SKBR-3/WT, SKBR-3/NT, and SKBR-3/PRLRKO (SG1, SG2, and SG3) cell lines. Upper panels show representative cell invasion experiment. Lower panels show quantification of results of four replicates of three independent experiments *P 0.012 (one-way ANOVA).

While the physiological role of PRL/PRLR in mammary epithelial cellular differentiation is well known, we aimed here to evaluate the impact of loss of PRLR expression in regulating plasticity and tumorigenesis of the breast cancer subtypes HR+ (MCF-7 cells) and HER2-E (SKBR-3 cells) using CRISPR/Cas9 technology. Multiple isoforms of the PRLR have been described to be encoded by a single gene located on chromosome 5 (ref. ^34^). To ensure proper disruption of receptor expression, we designed three different single-guide RNAs (gRNAs) to selectively target exons 5 (SG1) and 6 (SG2 and SG3), encoding part of the receptor extracellular domain shared by PRLR isoforms (Supplementary Fig. 1a). Proper indel mutations were assessed and quantified using the surveyor nuclease cleavage assay and showed proper cleavage of the PRLR gene with all gRNAs in both MCF-7 and SKBR-3 cells (30–43%) (Supplementary Fig. 1b, c). We then assessed the resulting physical and functional loss of PRLR. As seen in Supplementary Fig. 2a, b, all three MCF-7/PRLRKO cell lines and SKBR-3/PRLRKO cell lines showed loss significant (*P* < 0.0001) in PRLR protein expression by (~50–60%) in comparison to their respective control WT and NT cell lines. In addition, all MCF-7/PRLRKO and SKBR-3/PRLRKO cell lines showed loss of the ability of PRL to induce phosphorylation of the downstream PRLR-signaling molecule Stat5a/b (Fig. 1a and b, respectively). To begin to evaluate the effects of loss of PRLR expression on the tumorigenic potential of the HR+ MCF-7 and the HER2-E SKBR-3 cells, first we examined the effects of loss of PRLR expression on cell viability, migration, and invasion capacities using in vitro assays. As can be seen in Fig. 1c, d, all MCF-7/PRLRKO and SKBR-3/PRLRKO cell lines displayed significant (*P* < 0.0001) increase in cell viability in comparison to their corresponding control cell lines (~54% increase seen in the MCF-7 cell model and ~66% increase seen in the SKBR-3 cell model). Next, we performed wound closure assays to examine the migratory properties of the MCF-7/PRLRKO cell lines in comparison to the control groups. Our results revealed that while WT and NT cells showed limited cell migration activity, even after 72 h, all MCF-7/PRLRKO cell lines showed accelerated cellular migration capacity with complete wound closure by 72 h (*P* < 0.0001) (Fig. 1e). To obtain further insights into their invasive capacities we used trans-well Matrigel invasion assay. As shown in Fig. 1f, after 48 h, we did not detect any invading cells in the control groups, while under the same conditions all MCF-7/PRLRKO cell lines showed increased cellular invasive capacity (*P* < 0.0001). We then used similar approaches to investigate the effects of disruption of PRLR expression in the HER2-E SKBR-3 cells on cell migration and invasion capacities. As shown in Fig. 1g, h, SKBR-3/PRLRKO cell lines showed an increase in both cell migration (*P* 0.0054) and invasion (*P* 0.012) capacities in comparison to the control groups. Together these results indicate that loss of PRLR expression in HR+ and HER2-E breast cancer cells results in increased survival, migratory, and invasive capacities, suggesting that PRLR expression endow breast cancer cells with a less aggressive phenotype, thereby limiting their tumorigenic properties.

### Loss of PRLR expression in HR**+** and HER2-E breast cancer cells alters their differentiation state and response to therapy

Breast cancer cells are known to be plastic in nature, able to convert to less differentiated and more aggressive phenotypes during disease progression. Having shown that loss of PRLR expression in both HR+ and HER2-E breast cancer cells augmented their aggressive phenotype, we next investigated whether it could also modulate their differentiation state. For this, we examined expression of ER and HER2 proteins, two known clinical breast cancer biomarkers defining the HR+ and the HER2-E subtypes, respectively, as well as several other markers defining various breast cancer differentiation states including luminal, epithelial, basal, and mesenchymal phenotypes.

#### HR+ cells

**Fig. 2:**
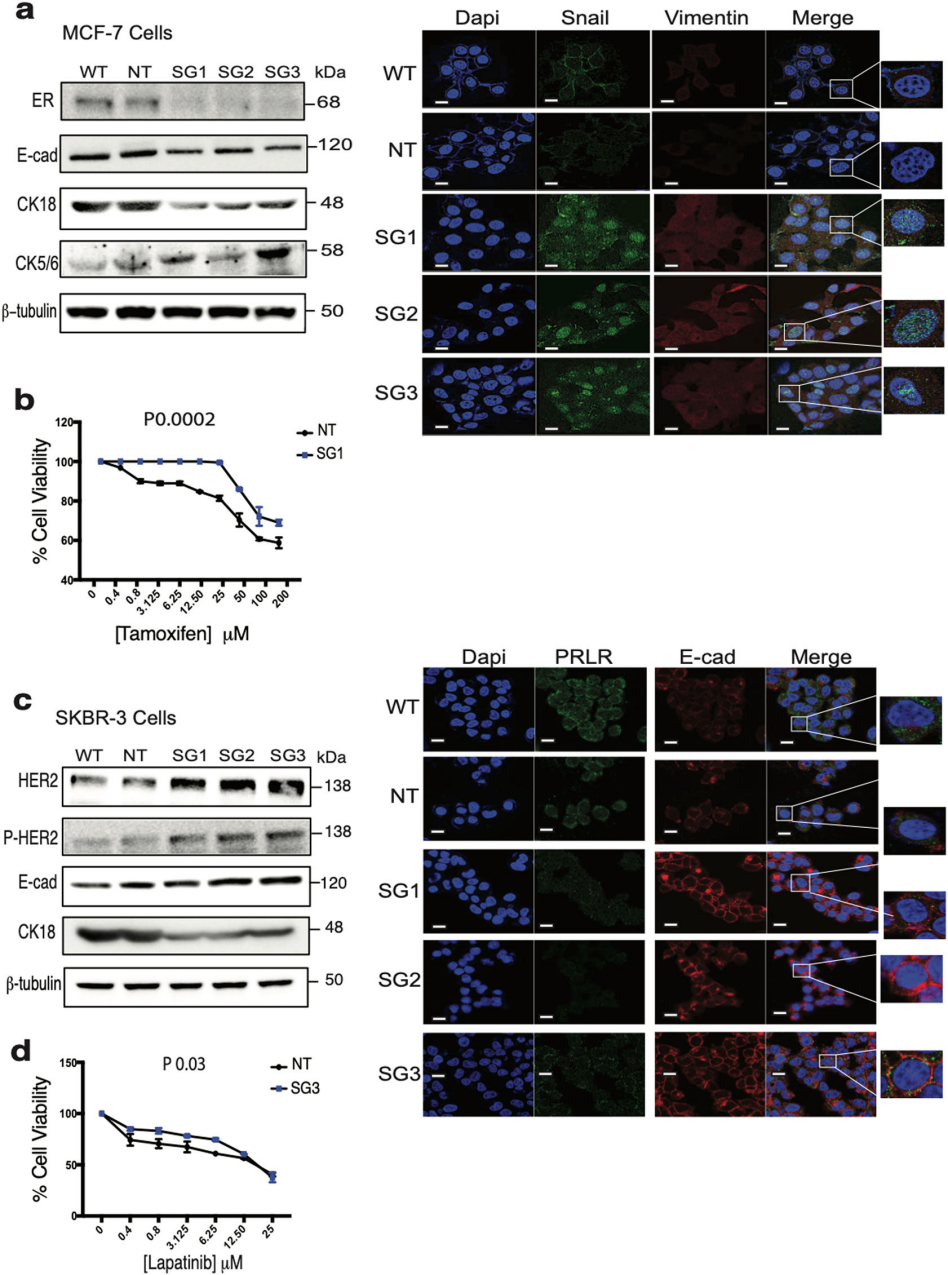
Loss of PRLR expression breast cancer cells altered their molecular features and resulted in reduced sensitivity to therapy. **a** Left panel, MCF-7/WT, MCF-7/NT, and MCF-7/PRLRKO (SG1, SG2, and SG3) cell lines were lysed and western blotting was carried out using antibodies against ER, E-cad, CK18, CK5/6, and β-tubulin. Right panel, confocal immunofluorescence images of snail (green), vimentin (red), and nucleus (DAPI) (blue) of MCF-7/WT, MCF-7/NT, and MCF-7/PRLRKO (SG1, SG2, and SG3) cell lines. Scale bar, 10 μm. **b** Proliferation SRB assays were performed on MCF-7/NT and MCF-7/PRLRKO (SG1) cell lines following 3 days treatment with tamoxifen (0–200 μM). Results are expressed as mean ± SEM of four replicates of three independent experiments ***P 0.0002 (two-way ANOVA). **c** Left panel, SKBR-3/WT, SKBR-3/NT, and SKBR-3/PRLRKO (SG1, SG2, and SG3) cell lines were lysed and western blotting was carried out using antibodies against HER2, Phospho-HER2, E-cad, CK18, and β-tubulin. Right panel, confocal immunofluorescence images of PRLR (green), E-cad (red), and nucleus (DAPI) (blue) of SKBR-3/WT, SKBR-3/NT, and SKBR-3/PRLRKO (SG1, SG2, and SG3) cell lines. Scale bar, 10 μm. **d** Proliferation SRB assays were performed on SKBR-3/NT and SKBR-3/PRLRKO (SG3) cell lines following 3 days treatment with lapatinib (0–25 μM). Results are expressed as mean ± SEM of triplicates of four independent experiments *P 0.03 (two-way ANOVA).

As shown in Fig. 2a (left panel) and Supplementary Fig. 3a, loss of PRLR expression in the HR+ MCF-7 cells resulted in loss of ER protein expression in comparison to the control groups. In addition, MCF-7/PRLRKO cell lines also showed loss of expression of both E-cad (epithelial marker) and cytokeratin-18 (CK18) (luminal marker) compared to the control groups (Fig. 2a (left panel) and Supplementary Fig. 3a). We next examined expression of the basal marker, cytokeratin 5/6 (CK5/6). As shown in Fig. 2a (left panel) and Supplementary Fig. 3a, MCF-7/PRLRKO cell lines showed higher CK5/6 protein expression compared to the control groups. To further examine the impact of loss of PRLR expression on the differentiation state of the HR+ MCF-7 breast cancer cells, we used immunofluorescence confocal microscopy to assess the expression pattern of the mesenchymal and EMT markers, vimentin and the transcription factor Snail. As shown in Fig. 2a (right panel), while MCF-7/WT and MCF-7/NT cell lines exhibited no or negligible expression of these mesenchymal markers, expression of vimentin and Snail was restored in all MCF-7/PRLRKO cell lines. Having shown that loss of PRLR expression led to a significant decrease in ER expression, we next sought to investigate whether PRLR expression could also regulate their response to hormonal therapy (i.e. tamoxifen). For this, MCF-7/NT and MCF-7/PRLRKO cell lines were subjected to tamoxifen treatment (0–200 μM) for 3 days and assessed for cell viability using sulforhodamine B (SRB) assay. As shown in Fig. 2b, MCF-7/PRLRKO cells showed a significant (*P* < 0.0002) reduction in sensitivity to tamoxifen as compared to control MCF-7/NT cells. Together, these results indicate that loss of PRLR expression in luminal/HR+ MCF-7 breast cancer cells results in their dedifferentiation to a basal-mesenchymal-like phenotype and further reduce their sensitivity to anti-hormonal therapy.

#### HER2-E cells

Next, we assessed the effects of loss of PRLR expression in modulating HER2 expression levels, the biomarker of HER2-E breast cancer subtype. As shown in Fig. 2c (left panel) and Supplementary Fig. 3b, SKBR-3/PRLRKO cell lines showed augmented levels of HER2 protein and activity (phospho-HER2) in comparison to the control groups. Interestingly, SKBR-3/PRLRKO cell lines also displayed gain in E-cad expression (Fig. 2c (left panel) and Supplementary Fig. 3b). Immunofluorescence analyses of SKBR-3/WT, SKBR-3/NT, and SKBR-3/PRLRKO cell lines for PRLR and E-cad confirmed the loss of expression of the PRLR in KO cell lines in comparison to control (WT and NT) cell lines and showed the enhanced expression of E-cad in SKBR-3/PRLRKO cell lines (Fig. 2c (right panel)). Next, to investigate whether loss of PRLR expression in HER2-E breast cancer cells affect their luminal differentiation state, we examined expression of the luminal marker CK18. Loss of PRLR expression in SKBR-3 cells led to a loss of CK18 expression (Fig. 2c, left panel and Supplementary Fig. 3b). Next, we investigated whether loss of PRLR expression modulates the sensitivity of HER2-E SKBR-3 cells to targeted therapy. For this, we assessed cell viability in response to treatment with lapatinib, a dual EGFR/HER2 kinase inhibitor^35^. SKBR-3/NT and SKBR-3/PRLRKO (SG3) cells were incubated with different concentrations (0–25 μM) of lapatinib for 3 days before assessing cell viability using SRB assay. As shown in Fig. 2d, lapatinib treatment was more effective in control SKBR-3/NT cells in comparison to SKBR-3/PRLRKO cells (*P* < 0.03), indicating that loss of PRLR expression leads to a marginal resistance to EGFR/HER2 kinase inhibitor treatment. Together, these results indicate that loss of PRLR expression in HER2-E breast cancer cells causes aberrant luminal and epithelial differentiation state with enhanced HER2 expression/activity and reduced sensitivity to HER2 targeted therapy. Collectively, these results reveal that loss PRLR expression alters the luminal and epithelial differentiation state of breast cancer cells in a subtype-dependent manner.

### Loss of PRLR expression promotes breast cancer stemness in a breast cancer subtype-dependent manner

**Fig. 3:**
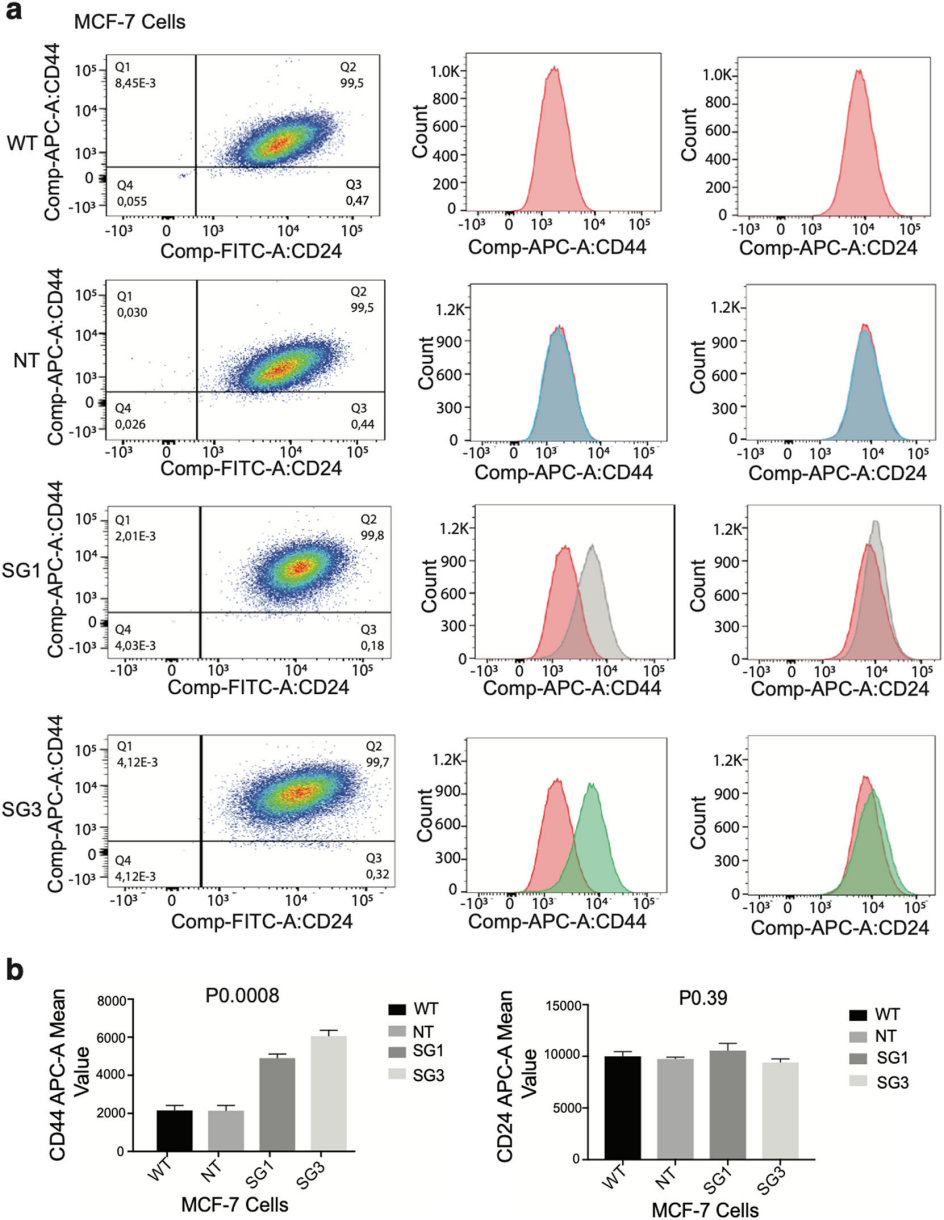
Interfering with PRLR expression in MCF-7 breast cancer cells enriched for CD44 BCSC marker expression. **a** Content of CD44+-BCSCs in MCF-7/WT, MCF-7/NT, and MCF-7/PRLRKO (SG1 and SG3) cell lines were determined by flow cytometry. Representative images of dot plot (left panels) and histograms of CD44 (middle panels) and CD24 (right panels) are shown. **b** Quantification analysis of BCSCs markers CD44 and CD24 in MCF-7/WT, MCF-7/NT, and MCF-7/PRLRKO (SG1 and SG3) cell lines expressed as mean ± SEM of duplicates of three independent experiments (CD44−APC-A, ***P 0.0008) and (CD24−FITC-A, *P* 0.39) (one-way ANOVA).

**Fig. 4:**
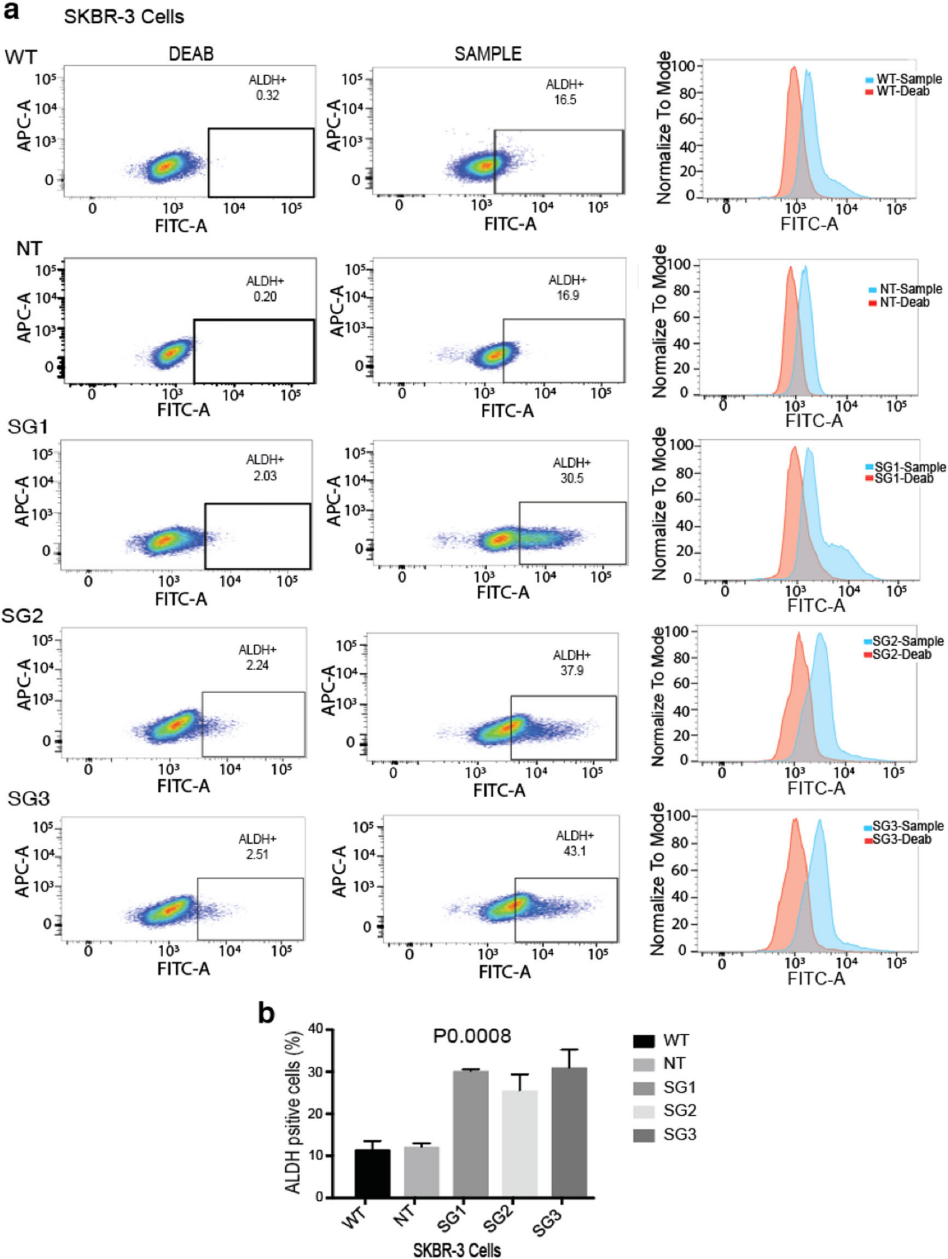
Loss of PRLR in SKBR-3 breast cancer cells augmented breast cancer stem-like cells markers expression. **a** ALDH+ BCSCs were determined using flow cytometry in SKBR-3/WT, SKBR-3/NT, and SKBR-3/PRLRKO (SG1, SG2, and SG3) cell lines. Left panels, representative images of dot plots (DEAB treated) and samples. Right panels show histograms. **b** Quantification analysis of ALDH+ BCSCs in SKBR-3/WT, SKBR-3/NT, and SKBR-3/PRLRKO (SG1, SG2, and SG3) cell lines expressed as mean ± SEM of duplicates of three independent experiments, ***P 0.0008 (one-way ANOVA).

We showed that loss of PRLR expression in HR+ and HER2-E cells caused dedifferentiation in their molecular phenotype and increased their survival, migratory, and invasive properties, all of which are indicators of the acquisition of a cancer stem-like phenotype. Basal-like breast tumors, which display mesenchymal and invasive properties, are enriched in CD44+CD24−/low BCSCs whereas HER-2E tumors, mostly contain ALDH+ characterized by epithelial highly proliferative and metastatic BCSCs^36–39^. CSCs can grow in low attachment conditions and form tumorspheres in suspension. Therefore, next we examined the in vitro tumorsphere formation capacity (TFC) of MCF-7/PRLRKO and SKBR-3/PRLRKO cell lines in comparison to their respective control cell lines. As shown in Supplementary Fig 4a, b, loss of PRLR expression in both MCF-7 and SKBR-3 cells resulted in increase in their TFC (*P* < 0.0001), under both growth conditions, compared to control cells. Interestingly, whereas MCF-7 cells formed tumor sphere-shaped colonies in suspension, SKBR-3 cells formed islands-shaped epithelial sheets when grown in suspension. To further identify the specific BCSC populations being affected by loss of PRLR expression in MCF-7 and SKBR-3 cells, we first examined the expression of the cell surface stem cell markers CD44 and CD24 by flow cytometry. As can be seen in Fig. 3a, b, MCF-7/WT and MCF-7/NT cell lines mostly showed CD44+/CD24+ double-positive cell population. Interestingly, MCF-7/PRLRKO cell lines (SG1 and SG3 examined) showed a significant (*P* 0.0008) increase in CD44 expression with no change (*P* 0.39) in CD24 expression in comparison to the control groups. On the other hand, we did not find any significant changes in CD44 or CD24 expression in SKBR-3/PRLRKO cell lines in comparison to the control groups (Supplementary Fig. 5a–c). Next, we assayed for the presence of the ALDH+ BCSC population using ALDEFLUOR assay. Each cell sample was compared to its own negative control sample in which cells were treated with the ALDH inhibitor *N*,*N*-diethylamino benzaldehyde (DEAB)^40^. As can be seen in Supplementary Fig. 6a, b, MCF-7 cells showed limited number of ALDH+ BCSC subpopulation (~3%) and loss of PRLR expression did not show significant (*P* 0.07) change in this population. In contrast, SKBR-3 wild-type cells have ~16% ALDH+ BCSC population (Fig. 4a, b). Indeed, all SKBR-3/PRLRKO cell lines showed a significant (*P* 0.0008) more than two-fold increase in ALDH+ BCSC population in comparison to the control groups. It is also noteworthy to note that SKBR-3/PRLRKO cell lines exhibited higher cell population of DEAB-resistant ALDH+ cells (~2%) in comparison to SKBR-3 WT and NT cell lines contained less than 0.5% of this cell population (Fig. 4a). Together these results indicate that PRLR expression modulates breast cancer stemness in a subtype context-dependent manner and acts as a safeguard against enrichment of these aggressive BCSC populations in both HR+ and HER-2E molecular subtypes.

### Loss of PRLR in HR+ breast cancer cells initiates tumor development of mesenchymal and basal phenotype

**Fig. 5:**
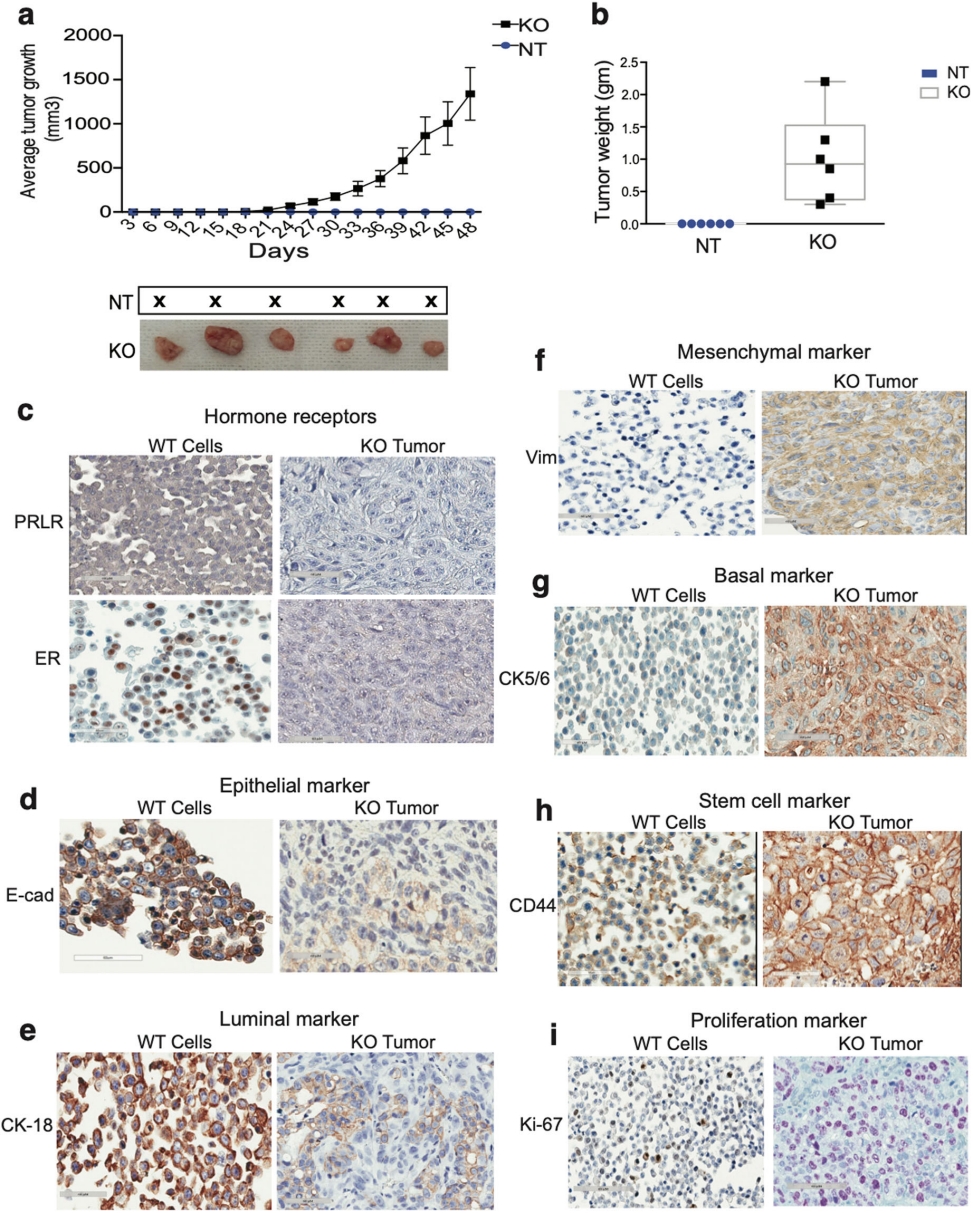
Loss of PRLR expression in MCF-7 breast cancer cells generated mesenchymal and basal-like tumors in vivo. **a** Upper panels, tumor volume measurements of xenografts of MCF-7NT and MCF-7/PRLRKO followed for a period of 48 days, *P 0.012 (Student’s *t*-test). Lowe panel, images of MCF-7/PRLRKO tumor-xenografts. **b** Tumor weight measurements of xenografts of MCF-7/NT and MCF-7/PRLRKO, **P 0.0052 (Student’s *t*-test). **c** Immunohistochemical staining of hormone receptors (PRLR and ER) in MCF-7/WT cells and MCF-7/PRLRKO tumors (×40). **d** Immunohistochemical staining of the epithelial marker E-cad in MCF-7/WT cells and MCF-7/PRLRKO tumors (×40). **e** Immunohistochemical staining of the luminal marker CK18 in MCF-7/WT cells and MCF-7/PRLRKO tumors (×40). **f** Immunohistochemical staining of the mesenchymal marker vimentin in MCF-7/WT cells and MCF-7/PRLRKO tumors (×40). **g** Immunohistochemical staining of the basal marker CK5/6 in MCF-7/WT cells and MCF-7/PRLRKO tumors (×40). **h** Immunohistochemical staining of the stem cell marker CD44 in MCF-7/WT cells and MCF-7/PRLRKO tumors (×40). **i** Immunohistochemical staining of the proliferative marker Ki67 in MCF-7/WT cells and MCF-7/PRLRKO tumors (×40).

Next, using immune deficient cell line-derived xenograft mouse models (CDX), we assessed the effects of loss of PRLR expression in breast cancer cells on tumor development in vivo. For this we performed mammary fat pad orthotopic transplantation of MCF-7/NT and MCF-7/PRLRKO (SG1) cell lines. MCF-7 cells do not form tumors in xenograft mouse models, unless supplemented with estrogen^41^. Therefore, to examine exclusively the effect of loss PRLR expression on tumor development in vivo we chose not to supplement the mice with estrogen. Interestingly, as shown in Fig. 5a, b and Supplementary Fig. 7a, b, all mice transplanted with MCF-7/PRLRKO cells developed tumors of variable sizes and weights within 3 weeks. In contrast and as expected none of the mice transplanted with MCF-7/NT cells developed tumors. To characterize the type of MCF-7/PRLRKO tumors that were generated, using immunohistochemistry we examined expression of various biomarkers in these tumors in comparison to control MCF-7 wild-type cells grown in vitro (due to the fact that no tumors were generated in vivo when using MCF-7/NT cells). As expected, in contrast to MCF-7/WT cells showing PRLR staining, MCF-7/PRLRKO tumors showed no expression of PRLR (*P* 0.0007) (Fig. 5c and Supplementary Fig. 7c). Next, we examined the effects of loss of PRLR expression on the expression of different biomarkers of HR+ breast cancer subtype. While MCF-7/WT cells showed nuclear ER expression, MCF-7/PRLRKO tumors showed significant loss of ER expression (*P* 0.0028) (Fig. 5c and Supplementary Fig. 7d). Moreover, while MCF-7/WT cells showed staining of E-cad and CK18, MCF-7/PRLRKO tumors showed loss in expression of both markers (*P* 0.0019 and *P* 0.0016 respectively) (Fig. 5d, e and Supplementary Fig. 7e, f). Next we investigated whether MCF-7/PRLRKO tumors gained mesenchymal and basal phenotypes. As shown in Fig. 5f–h and Supplementary Fig. 7g–i, in contrast to control MCF-7/WT cells, MCF-7/PRLRKO tumors did show gain in the expression of vimentin (*P* 0.004), CK5/6 (*P* 0.0034) and CD44 (*P* 0.029). Next, we examined the proliferative capacity of MCF-7/PRLRKO tumors in comparison to MCF-7/WT cells. As shown in Fig. 5i and Supplementary Fig. 7j, MCF-7/PRLRKO tumors showed increased staining of Ki67 (*P* 0.00169) in comparison to MCF-7 wild-type cells. Altogether these results demonstrate that loss of PRLR expression in HR+ breast cancer cells generates de-differentiated mesenchymal/basal-like proliferative tumors in vivo.

### Loss of PRLR expression in HER2-E breast cancer cells enhanced tumor development of epithelial HER2-E phenotype

**Fig. 6:**
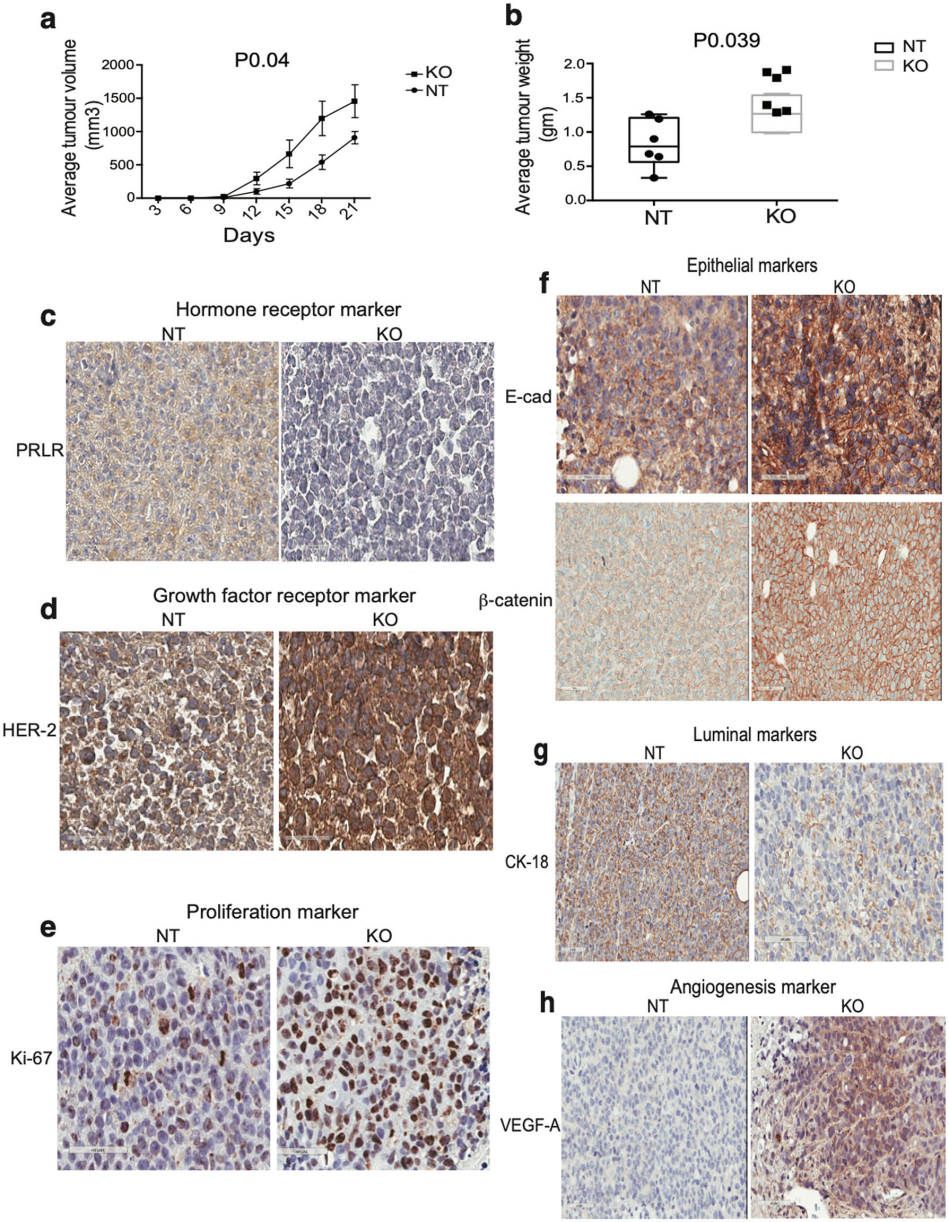
Loss of PRLR expression in SKBR-3 breast cancer cells accelerated tumor development showing epithelial, proliferative, and highly vascular phenotype with augmented HER-2 expression in vivo. **a** Measurements of tumor volume of xenografts of SKBR-3/NT and SKBR-3/PRLRKO followed for a period of 21 days, *P 0.04 (Student’s *t*-test). **b** Measurements of tumor weight of xenografts of SKBR-3/NT and SKBR-3/PRLRKO, *P 0.039 (Student’s *t*-test). **c** Immunohistochemical staining of PRLR in SKBR-3/NT and SKBR-3/PRLRKO tumor (×40). **d** Immunohistochemical staining of HER-2 in SKBR-3/NT and SKBR-3/PRLRKO tumor (×40). **e** Immunohistochemical staining of the proliferative marker Ki67 in SKBR-3/NT and SKBR-3/PRLRKO tumor (×40). **f** Immunohistochemical staining of the epithelial markers, E-cad and β-catenin, in SKBR-3/NT and SKBR-3/PRLRKO tumor (×40). **g** Immunohistochemical staining of the luminal marker CK18 in SKBR-3/NT and SKBR-3/PRLRKO tumor (×40). **h** immunohistochemical staining of the angiogenesis marker VEGFA in SKBR-3/NT and SKBR-3/PRLRKO tumor (×40).

Next, we examined the effects of loss of PRLR expression on tumor development in vivo in HER2-E SKBR-3 CDX mouse models. Our findings revealed that SKBR-3/PRLRKO cell line formed significantly (*P* 0.04) larger tumors as indicated by tumor volume and weight than tumors of SKBR-3/NT cell line (Fig. 6a, b and Supplementary Fig. 8a, b). Furthermore, as indicated in Supplementary Fig. 8a, SKBR-3/PRLRKO tumors (5/6 mice) appeared to be irregular, highly vascular, and invasive into the surrounding local area (such as the thigh). Whereas tumors in the control group (SKBR-3/NT) appeared well encapsulated, less vascular with no invasion of the surrounding tissue. Next, we analyzed expression of several biomarker in the SKBR-3/PRLRKO tumors in comparison to SKBR-3/NT tumors using immunohistochemistry. As expected, tumors of SKBR-3/PRLRKO showed loss of PRLR expression (Fig. 6c and Supplementary Fig. 8c) (*P* 0.0028). Interestingly, we found increased expression of HER-2 (*P* 0.002) (Fig. 6d and Supplementary Fig. 8d) and increased expression of Ki67 (*P* 0.0002) (Fig. 6e and Supplementary Fig. 8e). We next examined the expression of various epithelial and luminal markers. As shown in Fig. 6f and Supplementary Fig. 8f, g, we found significant increase in expression of E-cad as well as β-catenin (*P* 0.034 and *P* 0.0133, respectively) in SKBR-3/PRLRKO tumors in comparison to control SKBR-3/NT tumors. On the other hand, we observed a significant loss in expression of CK18 (*P* 0.009) in SKBR-3/PRLRKO tumors in comparison to SKBR-3/NT tumors (Fig. 6g and Supplementary Fig. 8h). Having found increased vascularization within the tumors of the SKBR-3/PRLRKO xenograft group, we next examined VEGFA expression, a downstream target of active HER2 signaling^42^. As shown in Fig. 6h and Supplementary Fig. 8i, SKBR-3/PRLRKO tumors showed increase in expression of the vascular marker in comparison to SKBR-3/NT tumors (*P* 0.0014) implying that SKBR-3/PRLRKO tumors harbor increased angiogenic capacity. Together these results highlight that loss of PRLR expression in HER2-E breast cancer cells generates HER-2-driven aggressive epithelial, proliferative and highly vascular tumors in vivo.

### Loss of PRLR in HR+ MCF-7 and HER2-E SKBR-3 cells enhanced their metastatic capacities in vivo

**Fig. 7:**
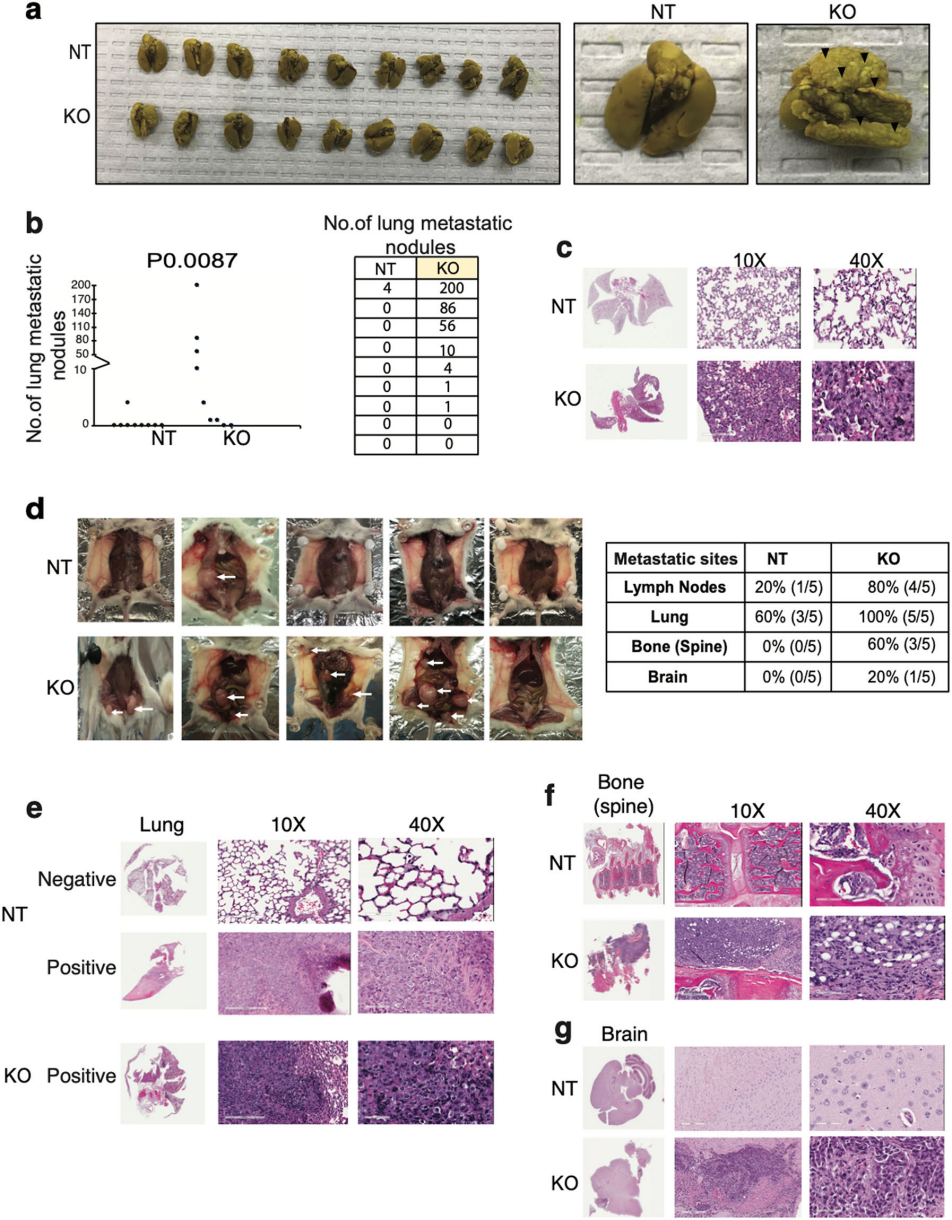
Breast cancer cells show high metastatic capacity upon loss of PRLR expression in preclinical mouse models. **a** Photos of lungs of NSG tail vein mouse models of MCF-7/NT and MCF-7/PRLRKO (left panel) and black arrow heads indicate macro-metastases present on representative lungs (right panel). **b** Quantification of lung nodules in MCF-7/NT and MCF-7/PRLRKO tail vein mouse models, **P 0.0078 (Student’s *t*-test). **c** Representative images of H&E staining of lung nodules of MCF-7/NT and MCF-7/PRLRKO tail vein mouse models (×10, ×40). **d** Left panel, photos of SKBR-3/NT and SKBR-3/PRLRKO NOD-SCID tail vein mouse models. White arrow heads indicate macro-metastases. Right panel, tabulation of the organs involved in extrapulmonary spread in SKBR-3/NT and SKBR-3/PRLRKO tail vein mouse models. **e–g** Representative images of H&E staining of lungs, bones, and brain metastases of SKBR-3/NT (left panel) and SKBR-3/PRLRKO (right panel) tail vein mouse models (×10, ×40).

Next, we examined the effects of loss of PRLR expression in both breast cancer cell models on their metastatic properties in vivo. One million cells of either control (NT) cell lines or their corresponding (PRLRKO) cell lines were injected into the tail vein of immunocompromised mouse and the mice were followed for a period of 4 weeks. Interestingly, 77% (7/9) of MCF-7/PRLRKO mice showed lung nodules formation with variable nodule numbers reaching up to 200 nodules per lung. In contrast, only 11% (1/9) of control MCF-7/NT mice developed pulmonary nodules (four nodules) (*P* 0.0078) (Fig. 7a, b). We next examined the effects of loss of PRLR expression on lung colonization and metastasis in vivo, using the HER2-E SKBR-3 model. As shown in Fig. 7d–g whereas 60% of control mice injected with SKBR-3/NT cells developed lung metastatic nodules, 100% of the mice injected with SKBR-3/PRLRKO cells developed metastases. Moreover, while only 20% of the control SKBR-3/NT mice showed lymph node involvement, mice injected with SKBR-3/PRLRKO cell line massively showed lymph node involvement (80%) and multi-organ metastases, including bone (60%) and brain (20%). Together, these results indicate that loss of PRLR expression in both breast cancer cell models fuels the generation of highly metastatic breast cancer cells.

Collectively, these results demonstrate that expression of PRLR in breast cancer derives and supports luminal and epithelial differentiation, guarding against aggressive tumor behavior. Consequently, upon loss of PRLR expression cancer cells dedifferentiation leads to increased tumor CSC content, further contributing to tumor heterogeneity, metastasis, and resistance to therapy. Altogether, these results suggest that the PRL/PRLR pathway can be further exploited for prognostic and therapeutic opportunities in breast cancer

## Discussion

In epithelial cancers such as breast cancer the fundamental links between cancer differentiation state and tumor heterogeneity, aggressivity and treatment failure has ignited interest in identifying differentiation-dependent actionable therapeutic targets^11^. The present study highlights that promoting both luminal and epithelial differentiation is crucial to restrict breast tumorigenesis. As illustrated in Supplementary Fig. 9, loss of PRLR expression in luminal and epithelial breast cancer subtypes altered their differentiation status promoting the complex multifaceted tumorigenic features of breast cancer including stemness, tumor development, metastasis, and resistance to therapies and positions PRL/PRLR pathway as a differentiation therapeutic target in breast cancer.

We found that suppressing PRLR expression in the luminal HR+ MCF-7 breast cancer cells caused loss of their luminal (ER and CK18 markers expression) phenotype but enhanced their basal (CK5/6 marker expression) and mesenchymal/stemness (vimentin and CD44 markers expression) phenocopying human basal-like breast cancers^43^. These results also agree with our previous findings where PRLR m-RNA expression was found to positively correlate with epithelial cell–cell adhesion and luminal differentiation metagenes but to negatively correlate with basal-like and mesenchymal-like (claudin low) metagenes^23^. Interestingly, the basal-like/mesenchymal molecular features of MCF-7/PRLRKO tumors are also reminiscent of mammary tumors generated by the loss of function of several tumor suppressors such as *BRCA1* gene^44,45^, *BRCA1* and *TP53* (refs. ^46–48^), and *TP53* and *RB1* (ref. ^49^). Indeed, these mammary tumors are aggressive showing spindle-cell/mesenchymal-like histology, grouped closely with human claudin-low TNBC. Interestingly, these mammary tumors exhibited low expression levels of PRLR as well as other luminal and epithelial differentiation markers such as (*Elf5*, *Muc1*), claudins, and cell adhesion markers (*Cdh1*, *Ocln)*, while showing elevated levels of EMT and BCSC markers. These results stress the critical role of PRLR expression in inducing/maintaining the differentiation state of HR+ breast cancer deriving luminal and epithelial differentiation and suppressing basal-mesenchymal and stemness features.

Notably, while loss of PRLR enhanced the tumorigenic and metastatic potential of both HR+ and HER2-E breast cancer cells, these effects appear to be mediated through distinct mechanisms. Indeed, loss of PRLR expression in both HR+ and HER2-E cells showed loss of luminal differentiation. However, in contrast to HR+/PRLRKO breast cancer cells, HER2-E/PRLRKO cells and xenograft tumors displayed enriched epithelial phenotype showing increased numbers of ALDH+-BCSC population. This particular phenotype represents aggressive, proliferative/metastatic HER2-E tumors associated with poor patient outcome^39,50,51^. These features have been linked to metastatic tumor cell collective migration and survival^52–56^ hence may explain the extrapulmonary metastatic colonizations observed in HER2-E/PRLRKO cells. These results also support the notion that loss of PRLR expression in HER2-E tumor cells dis-engages the luminal and epithelial differentiation programs. These results are in line with our previous findings showing that PRL treatment of HER2-E breast cancer cells suppressed their tumorigenic capacity and suppressed ALDH+-BCSC population^27^. Altogether, these results emphasize that maintaining the expression of PRLR in HER2-E breast cancer subtype abrogates stemness and aggressive tumorigenic features by ensuring combined luminal and epithelial differentiation.

Clinically, when comparing metastatic vs primary tumors, the HR+ subtype was found to be the least stable able to convert to hormone independency showing worst patient outcome^57^. These clinically challenging breast cancer cases are reminiscent of our findings where loss of PRLR expression in the HR+ MCF-7 cells switched them to a TNBC-like profile with high tumorigenic and metastatic capacities. Most HER2-E breast tumors maintain their phenotype during tumor progression to a metastatic disease^57,58^. Our results also implicate that loss of PRLR expression in HER2-E cells enhanced HER2-driven tumorigenesis, metastasis, and resistance to therapy. Thus, loss of PRLR expression can also be predictive of metastasis and resistance to targeted treatment. Altogether, the PRL/PRLR pathway being a major mammary terminal differentiation pathway, able to drive differentiation of breast cancer cells can thus be proposed as a potential therapeutic target in breast cancer. Further extended preclinical settings and clinical trials should pave the way for the establishment of a much promising therapy.

## Materials and methods

### Antibodies, plasmids, and reagents

Antibodies, chemicals, and reagents were obtained from Santa-Cruz, Abcam, Millipore Sigma, BD Biosciences, and Invitrogen. For detail information refer to Supplementary Materials and Methods.

### PRLR and non-targeting single-guide sequence annealing and molecular cloning

LentiCRISPRv2 was digested using *Esp*3I restriction enzyme, dephosphorylated using FastAP, agarose gel purified and extracted using QIAquick Gel Extraction Kit. For complete designing steps refer to Supplementary Materials and Methods^59,60^.

### Cell culture, cell lines authentication, and Lentiviral infection

Human breast cancer cells: SKBR-3 and MCF-7 cells were maintained in Dulbecco’s modified Eagle’s medium (DMEM) media (Multicell Invitrogen) containing 10% fetal bovine serum (FBS) (Multicell Invitrogen). For cell line authentication and lentiviral transduction refer to Supplementary Materials and Methods.

### Western blotting analysis and immunoprecipitation

Total protein lysates were obtained using RIPA lysis buffer. Thirty microgram proteins were loaded in the gel. Cell lysates were separated by electrophoresis and electrophoretically transferred to a nitrocellulose membrane. Western blots were probed with the relevant primary antibodies and secondary antibodies. For complete steps refer to Supplementary Materials and Methods.

### Immunofluorescence

Fixation process were performed of coverslips coated with cells in 4% paraformaldehyde for 15 min at room temperature, followed by permeabilization and staining with primary antibody followed by secondary antibody and DAPI for 1 h at room temperature. For detail information refer to Supplementary Materials and Methods.

### Immunohistochemistry and scoring

Immunohistochemical staining was performed using Thermo Fisher Scientific detection kit. For detail staining and scoring refer to Supplementary Materials and Methods^30,61–63^.

### MTT assay

In all, 1000 cell/well were seeded into a 96-well plate and grown in 10% FBS DMEM for 24 h. Then, cells were incubated with 3-(4,5-dimethyl-2-thiazolyl)- 2,5-diphenyl-2H-tetrazolium bromide (MTT) at 37 °C for 2 h. Nanodrop was used to measure the OD at 570 nm.

### Scratch assay

5 × 10^3^ cell/well were seeded on six-wells plate in 10% FBS DMEM and grown until reached confluency. A straight scratch was obtained by yellow pipette tip and scratch or wound was monitoring by taking picture using ImageJ software at 0, 24, 48, and 72 h for MCF-7 cell model and up to 48 h for SKBR-3 cell model.

### Invasion assay

1 × 10^5^ cell/well were seeded in a 24-wells plate HTS multi-well insert system coated with Matrigel in 2% FBS DMEM in the upper chamber and 10% FBS DMEM in the lower chamber. Invasion assays were performed for 48 h, invaded cells were fixed, stained with 0.2% crystal violet, and counted using five fields of triplicates for each experimental point and pictures were taken using ImageJ software.

### Tumorsphere assay

In all, 1000 cells/well of MCF-7/WT, MCF-7/NT, and MCF-7/PRLRKO (SG1, SG2, and SG3) cell lines and 500 cells/well of SKBR-3/WT, SKBR-3/NT, and SKBR-3/PRLRKO (SG1, SG2, and SG3) were seeded in a ultra-low attachment 24-well plate (Corning), and cultured in serum-free DMEM medium supplemented with 10 ng/ml EGF, 10 ng/ml bFGF, and 1× B27 (Invitrogen) or DMEM medium with 1% FBS as described previously^64^. The plate was incubated at 37 °C with 5% CO_2_ for 7 days, without moving the plate. Pictures of the tumorspheres were taken using NIS microscopy imaging software.

### ALDEFLOUR assay and flow cytometry analysis

The ALDEFLUOR kit was used to measure ALDH activity. For detail information refer to Supplementary Materials and Methods.

### Sulforhodamine B assays

Cells cultured in 96-well plates and treated or left untreated with either tamoxifen (0–200 μM) or lapatinib (0–25 μM). For detail information refer to Supplementary Materials and Methods.

### Animal models, mammary fat pad NOD-SCID mouse xenografts, and tail vein NSG mouse xenografts

All experimental animal work was performed in a specific- pathogen-free animal facility according to the guidelines and ethical regulations of the Research Institute, McGill University Health Centre approved animal used protocol (#2014-7492) in accordance with Canadian Council of animal care guidelines. For detail information refer to Supplementary Materials and Methods^41^.

### Statistical analysis

Statistical analysis was performed using GraphPad prism 6 software using Student’s *t*-test, one-way, ANOVA or two-way ANOVA analysis accordingly. Results were shown as mean ± SEM and *P* < 0.05 was considered as cut-off for significant association.

## Supplementary information

Supplementary Figure 1

Supplementary Figure 2

Supplementary Figure 3

Supplementary Figure 4

Supplementary Figure 5

Supplementary Figure 6

Supplementary Figure 7

Supplementary Figure 8

Supplementary Figure 9

Legends to Supplementary Figures

Supplementary Materials and Methods
