# Supplementary figures and images for: Prolactin receptor-driven combined luminal and epithelial differentiation in breast cancer restricts plasticity, stemness, tumorigenesis and metastasis
Source: Oncogenesis. 2021 Jan 14;10(1):10. doi: 10.1038/s41389-020-00297-5 (PMC7809050; doi:10.1038/s41389-020-00297-5)

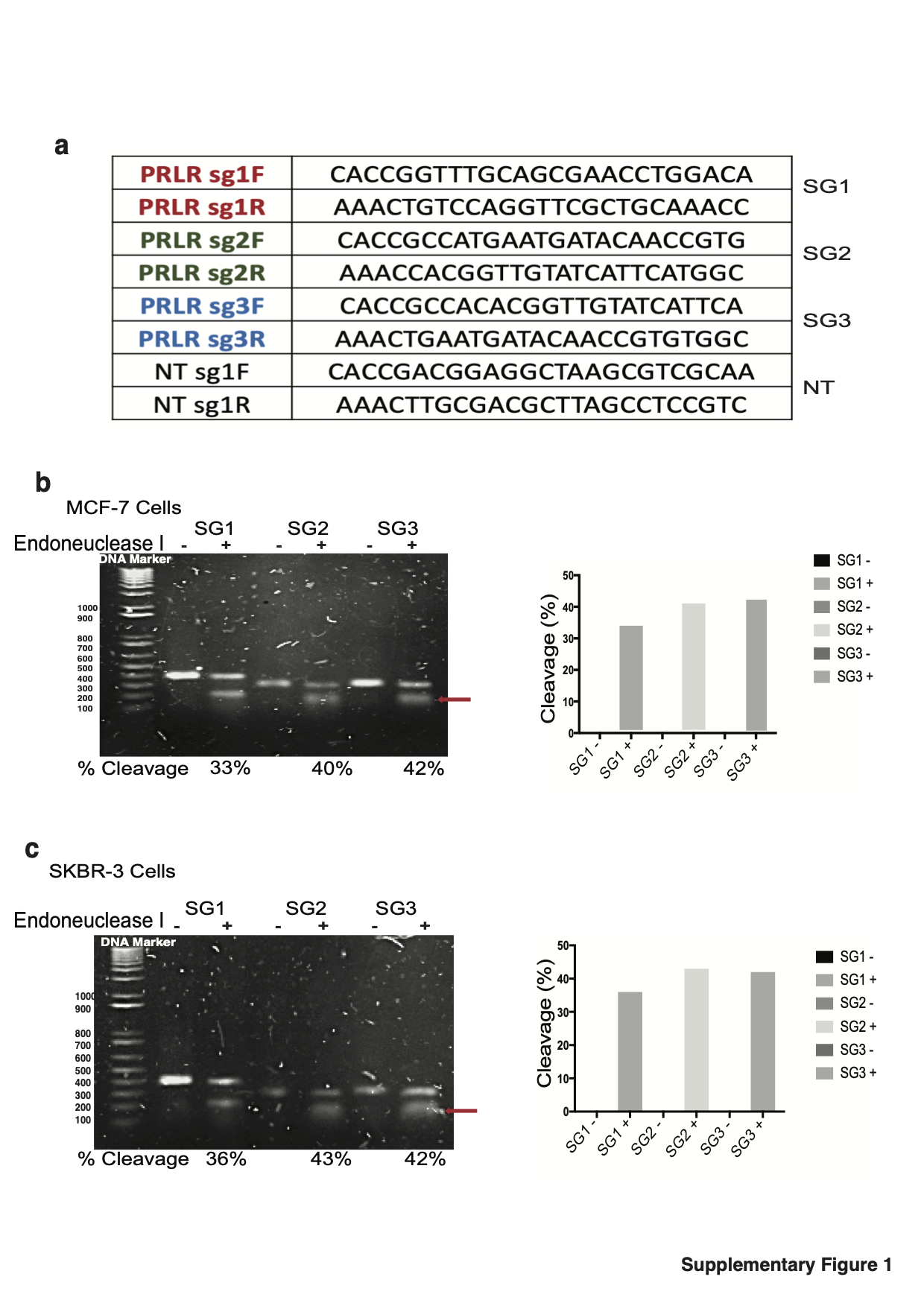

Supplement: Supplementary file 1 — Supplementary Figure 1 [file 41389_2020_297_MOESM1_ESM.tif]

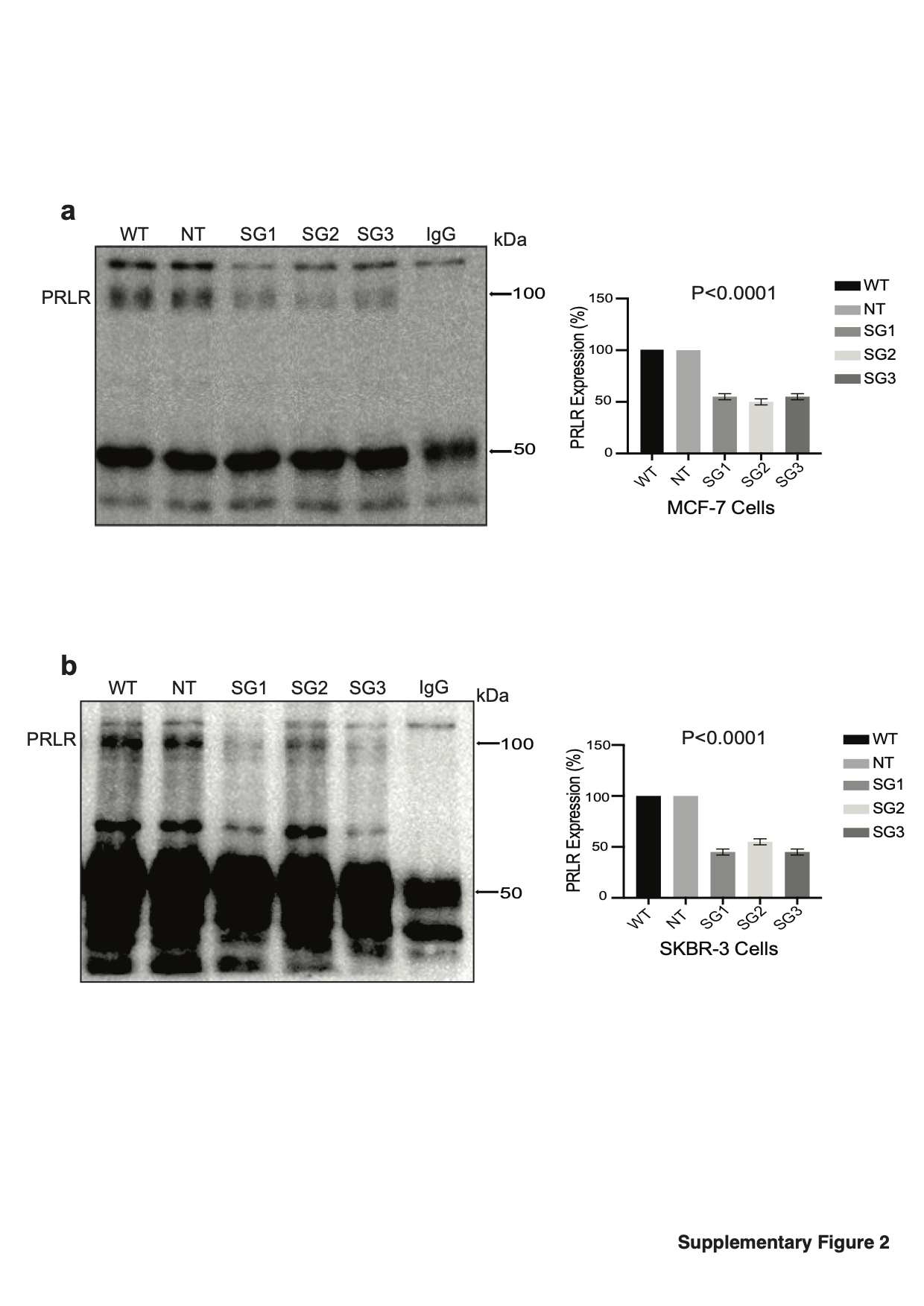

Supplement: Supplementary file 2 — Supplementary Figure 2 [file 41389_2020_297_MOESM2_ESM.tif]

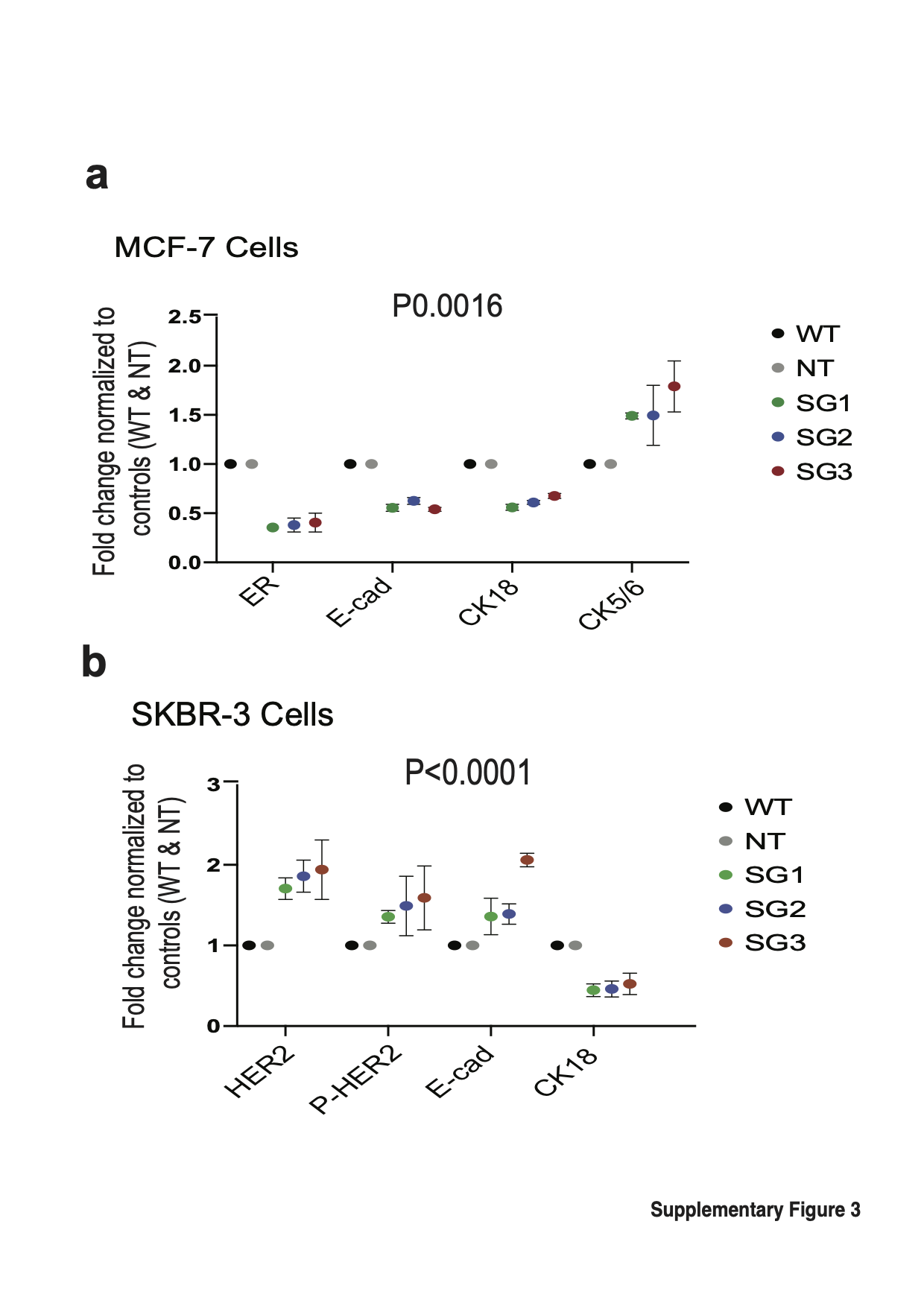

Supplement: Supplementary file 3 — Supplementary Figure 3 [file 41389_2020_297_MOESM3_ESM.tif]

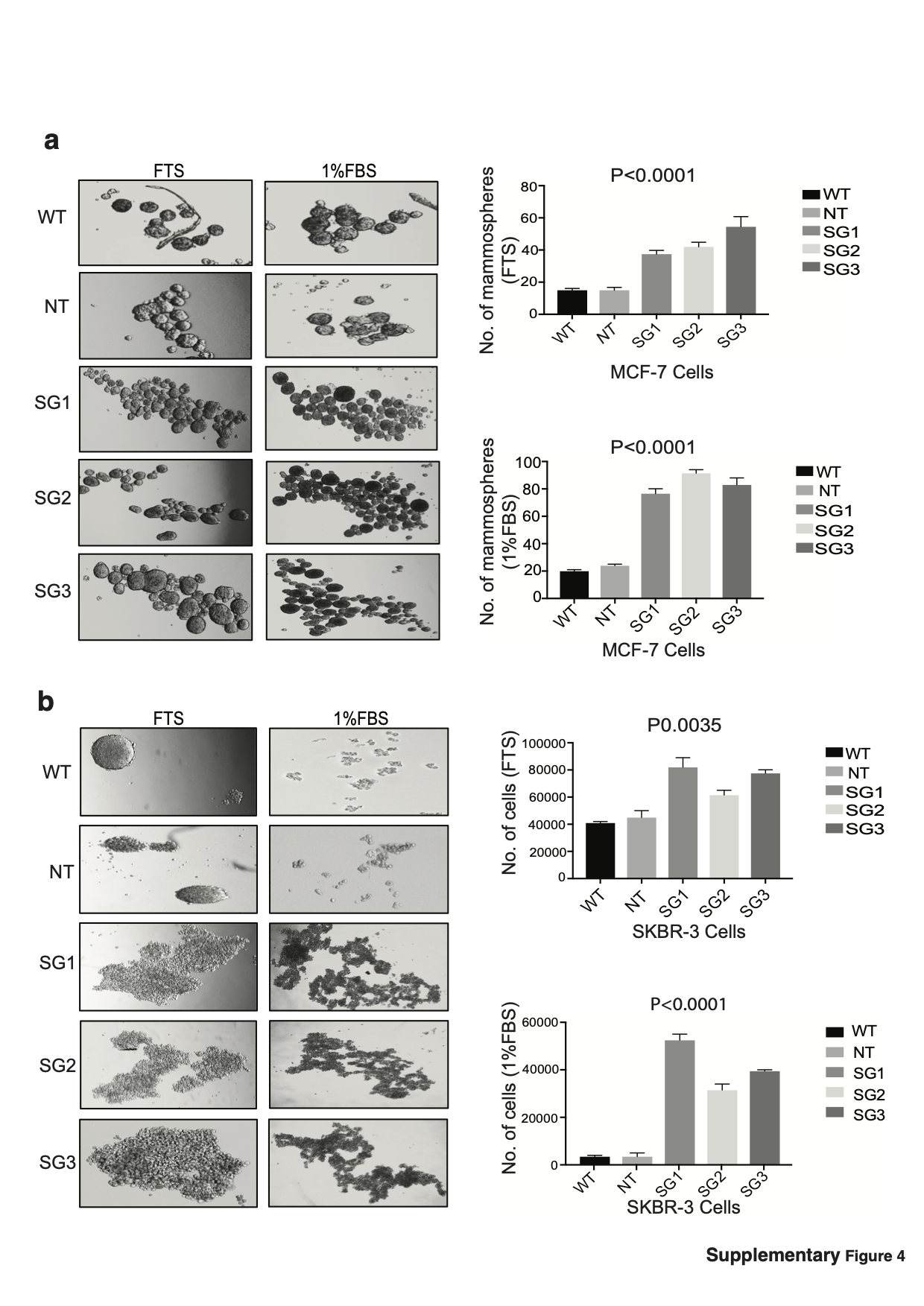

Supplement: Supplementary file 4 — Supplementary Figure 4 [file 41389_2020_297_MOESM4_ESM.tif]

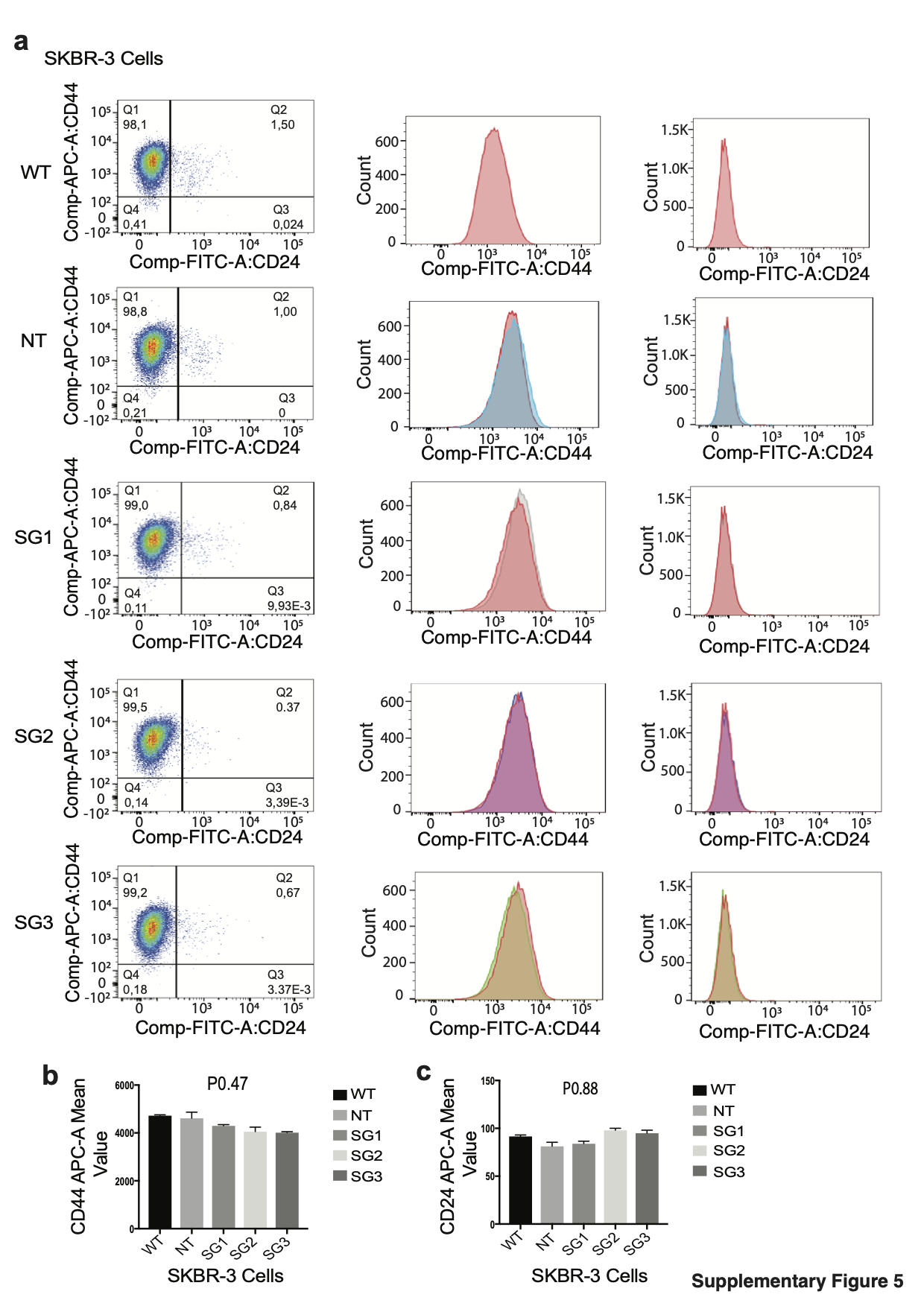

Supplement: Supplementary file 5 — Supplementary Figure 5 [file 41389_2020_297_MOESM5_ESM.tif]

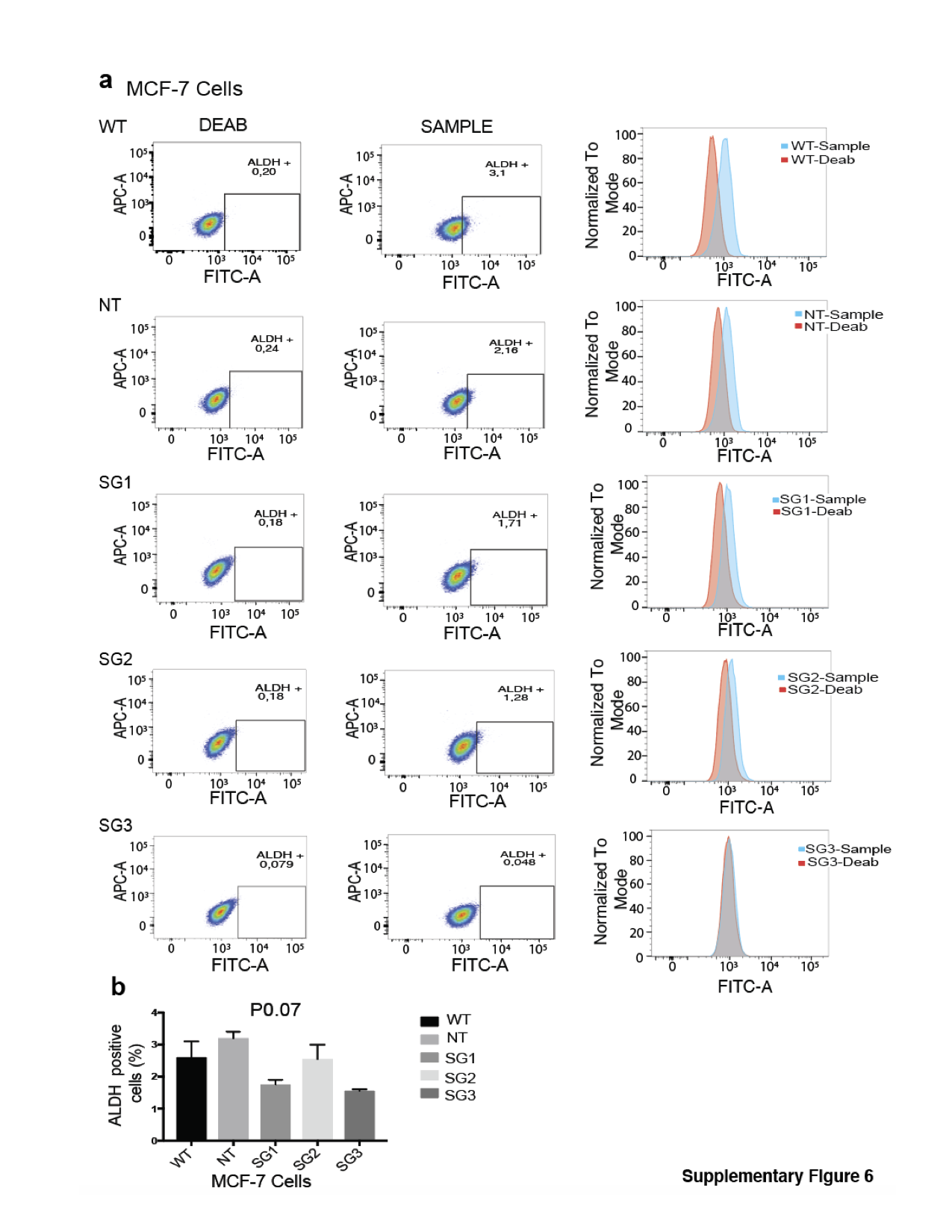

Supplement: Supplementary file 6 — Supplementary Figure 6 [file 41389_2020_297_MOESM6_ESM.tif]

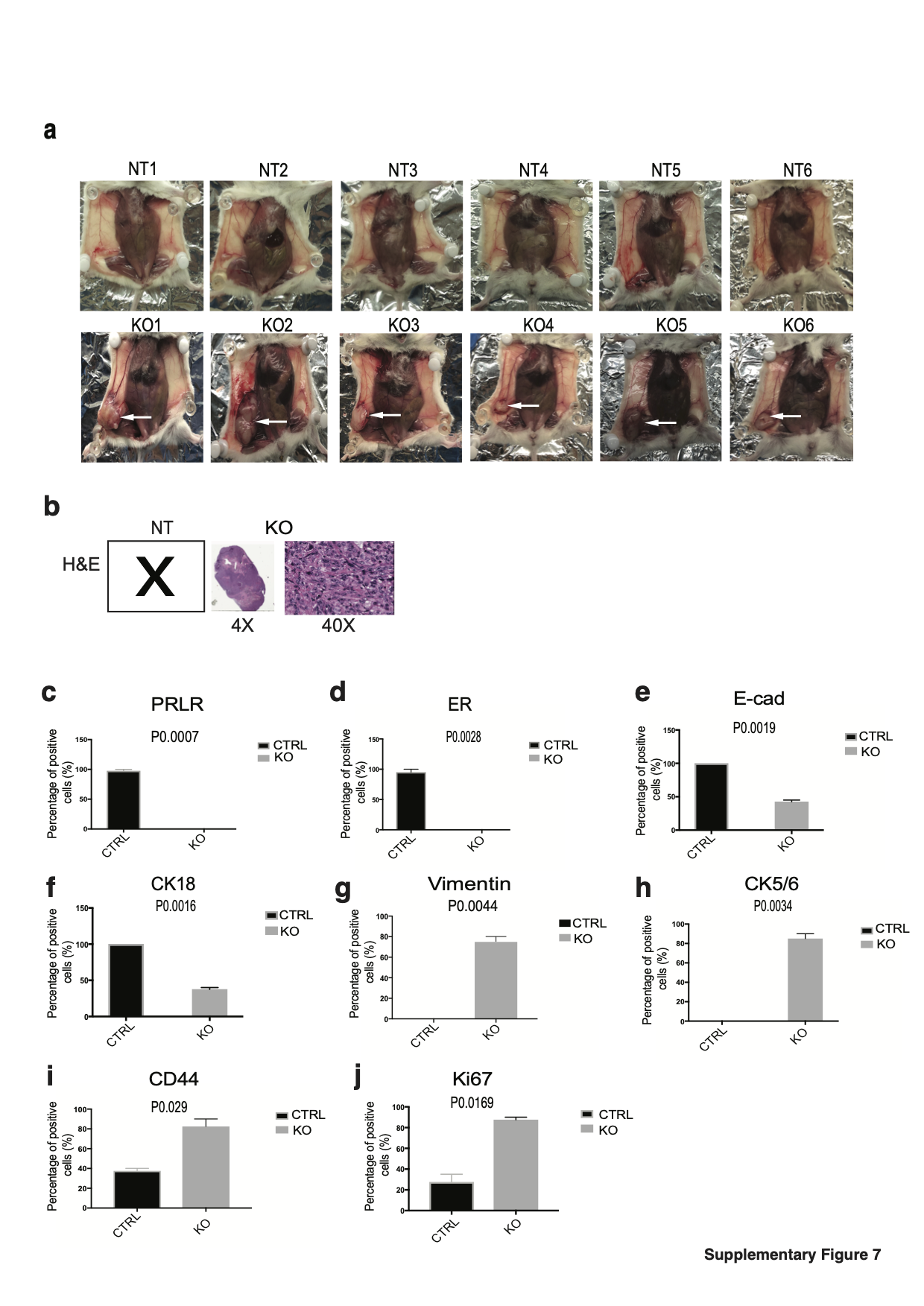

Supplement: Supplementary file 7 — Supplementary Figure 7 [file 41389_2020_297_MOESM7_ESM.tif]

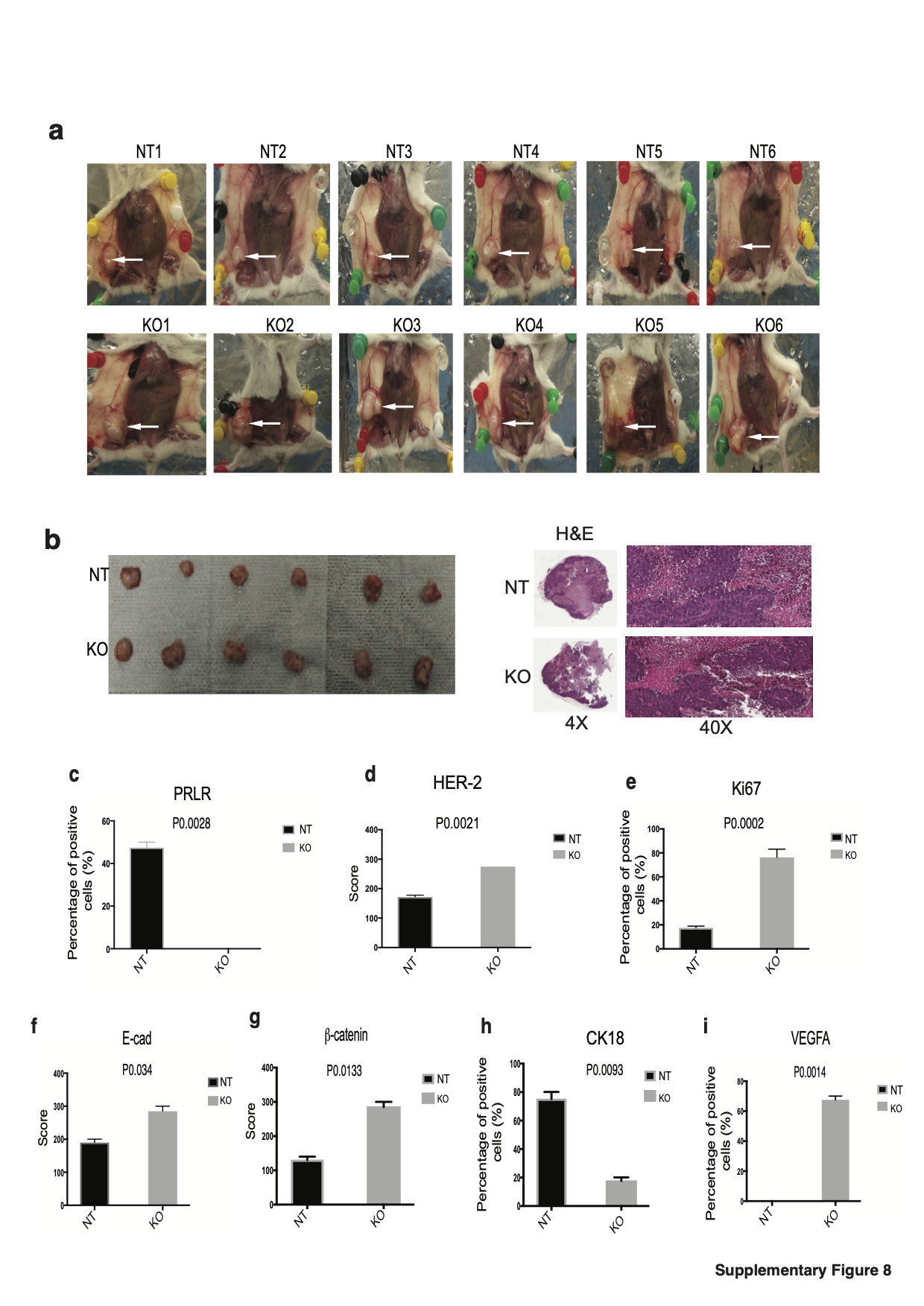

Supplement: Supplementary file 8 — Supplementary Figure 8 [file 41389_2020_297_MOESM8_ESM.tif]

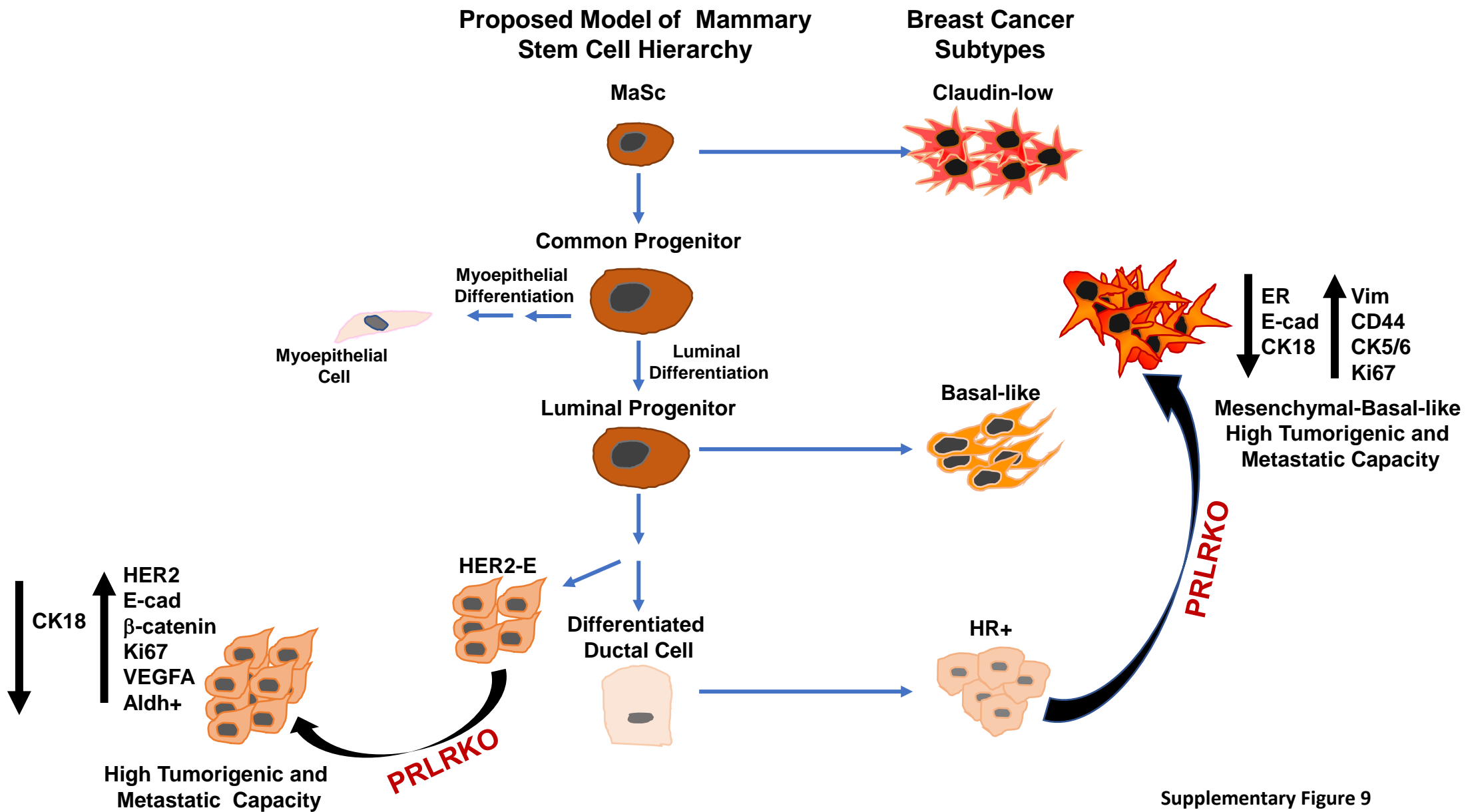

Supplement: Supplementary file 9 — Supplementary Figure 9 [file 41389_2020_297_MOESM9_ESM.pdf]
