# Supplementary material for: Prolactin receptor-driven combined luminal and epithelial differentiation in breast cancer restricts plasticity, stemness, tumorigenesis and metastasis
Source: Oncogenesis. 2021 Jan 14;10(1):10. doi: 10.1038/s41389-020-00297-5 (PMC7809050; doi:10.1038/s41389-020-00297-5)
Supplement: Supplementary file 10 — Legends to Supplementary Figures [file 41389_2020_297_MOESM10_ESM.docx]

**Legends to Supplementary Figures:**

**Supplementary Figure 1: Generation of CRISPR/Cas9 PRLR knockout cell lines**

**a.** Nucleic acid sequences of primers of sgRNA 1 (SG1) targeting PRLR exons 5, sgRNA 2 (SG2) and sgRNA 3 (SG3) targeting PRLR exon 6, and sgRNA 1 non-targeting (NT).

**b & c.** Left panels, agarose gel electrophoresis of nuclease-treated PCR products of MCF-7 and SKBR-3 PRLR knockout cell lines. Samples SG1 (lanes 1 & 2), SG2 (lanes 3 & 4) or SG3 (lanes 5 & 6) were re-annealed and untreated or treated with endonuclease I. Red arrows indicate the expected cleavage products. Right panels, quantification of the efficiency (%) of cleavage for each sgRNA is presented.

**Supplementary Figure 2: Loss of expression of the PRLR in CRISPR/Cas9 PRLR knockout cell lines**

**a & b.** Left panels, lysates of MCF-7/WT, MCF-7/NT and MCF-7/PRLRKO cell lines (SG1, SG2, and SG3) as well as of SKBR-3/WT, SKBR-3/NT and SKBR-3/PRLRKO cell lines (SG1, SG2, and SG3) were immunoprecipitated using a mouse monoclonal antibody against the PRLR or control normal mouse IgG using lysates of MCF-7/WT or SKBR-3/WT cells). Western blotting was carried out using a mouse monoclonal antibody against the PRLR. Right panels, quantitative analysis of PRLR expression normalized to the control WT & NT samples are shown as mean ± SEM of three independent experiments p<0.0001****(one-way ANOVA).

**Supplementary Figure 3: Loss of PRLR expression in breast cancer cells altered their differentiation characteristics**

**a**. Quantification of the expression (western blots) of different markers (as indicated in figure) in MCF-7/PRLRKO (SG1, SG2, and SG3) cell lines normalized to MCF-7/WT & MCF-7/NT control cell lines. The results are expressed as fold change of 3 independent experiments, p0.0016**(two-ways ANOVA).

**b**. Quantification of the expression (western blots) of different markers (as indicated in figure) in SKBR-3/PRLRKO (SG1, SG2, and SG3) cell lines normalized to SKBR-3/WT & SKBR-3/NT control cell lines. The results are expressed as fold change of 3 independent experiments, p<0.0001**** (two-ways ANOVA).

**Supplementary Figure 4: Loss of PRLR expression in breast cancer cells augmented their stemness phenotype**

**a & b**. Left panels, tumorsphere formation assays for a period of 7 days using full tumorsphere media or 1%FBS (as indicated) were performed using MCF-7/WT, MCF-7/NT and MCF-7/PRLRKO (SG1, SG2, and SG3) cell lines and SKBR-3/WT, SKBR-3/NT and SKBR-3/PRLRKO (SG1, SG2, and SG3) cell lines. Right panels, quantification of results expressed as mean ± SEM of duplicates of four independent experiments, p<0.0001****, p0.0035** (one-way ANOVA).

**Supplementary Figure 5: No change in expression levels of CD44 or CD24 stem-cell markers in SKBR-3 cells upon loss of PRLR expression**

**a**. left panels, dot plots and right panels, histograms of flow cytometry analysis of CD44 and CD24 expression in SKBR-3/WT, SKBR-3/NT and SKBR-3/PRLRKO (SG1, SG2, and SG3) cell lines.

**b & c**. Quantification of CD44 and CD24 expression levels in SKBR-3/WT, SKBR-3/NT and SKBR-3/PRLRKO (SG1, SG2, and SG3) cell lines expressed as mean ± SEM of duplicates of three independent experiments (CD-44-APC-A, p 0.47) and (CD24-FITC-A, p 0.88) (one-way ANOVA).

**Supplementary Figure 6: No change in ALDH+ BCSC population in MCF-7 cells upon loss of PRLR expression**

**a**. ALDH+ BCSCs were determined using flow cytometry in MCF-7/WT, MCF-7/NT and MCF-7/PRLRKO (SG1, SG2 and SG3) cell lines. Left panels, representative images of dot plots (DEAB treated) & samples). Right panels show histograms.

**b**. Quantification of ALDH+ BCSCs in MCF-7/WT, MCF-7/NT and MCF-7/PRLRKO (SG1, SG2 and SG3) expressed as mean ± SEM of duplicates of three independent experiments, p 0.07 (one-way ANOVA).

**Supplementary Figure 7: Loss of PRLR expression in MCF-7 breast cancer cells generated mesenchymal/basal tumors *in vivo***

**a**. Images of MCF-7/NT (upper panel) and MCF-7/PRLRKO (SG1) (lower panel) of NSG mice xenografts. White arrow heads indicate the tumors.

**b**. Representative H & E histology image of xenograft MCF-7/PRLRKO tumor (4X, 40X). X refers to MCF-7/NT samples where no xenograft tumors were developed.

**c-k**. Quantification of IHC analyses of MCF-7/WT cells & MCF-7/PRLRKO xenograft tumors of different markers: PRLR (p0.0007***), ER (p0.0028**), E-cad (p0.0019**), CK18 (p0.0016**), Vim (p0.0044**), CD44 (p0.029*), Ki67 (p0.016*), HER-2 (p0.0021**), CK5/6 (p0.0034**). Results are expressed as mean ± SEM of two independent experiments (Student's t-test).

**Supplementary Figure 8: Loss of PRLR expression in SKBR-3 breast cancer cells augmented HER-2 driven epithelial tumorigenesis *in vivo***

**a**. Images of SKBR-3/NT (upper panel) and SKBR-3/PRLRKO tumors (lower panel) of NSG mice xenografts. White arrow heads indicate the tumors.

**b**. Representative H & E histological image of xenograft tumors of SKBR-3/NT & SKBR-3/PRLRKO (4X, 40X).

**c-h**. Quantification of expression of biomarkers by IHC in SKBR-3/NT& SKBR-3/PRLRKO xenograft tumors: PRLR (p0.0028**), E-cad (p0.034*), β-catenin (p0.0133*), CK18 (p0.0093**), HER2 (p0.0021**), Ki67 (p0.0002***), and VEGFA (p0.0014**). Results are expressed as mean ± SEM of two independent experiments (Student's t-test).

**Supplementary Figure 9: PRLR expression regulates the differentiation state of breast cancer cells limiting stemness and tumorigenesis:** targeting prolactin receptor causes breast cancer subtype dependent dedifferentiation promoting tumor development, metastatic spread and resistance to therapy.
