# Supplementary material for: Prolactin receptor-driven combined luminal and epithelial differentiation in breast cancer restricts plasticity, stemness, tumorigenesis and metastasis
Source: Oncogenesis. 2021 Jan 14;10(1):10. doi: 10.1038/s41389-020-00297-5 (PMC7809050; doi:10.1038/s41389-020-00297-5)
Supplement: Supplementary file 11 — Supplementary Materials and Methods [file 41389_2020_297_MOESM11_ESM.docx]

**Supplementary Materials and Methods**

**1. Antibodies, plasmids and reagents**

Anti-PRLR mouse monoclonal antibody (D-7, Santa-Cruz #sc-377098), anti-PRLR rabbit polyclonal antibody (H-300, Santa-Cruz #sc- 20992), anti-Cytokeratin 18 rabbit monoclonal antibody (Abcam # ab133263), anti-Cytokeratin 5/6 mouse monoclonal antibody (Millipore Sigma # MAB1620), anti-E-cadherin (ECAD) mouse monoclonal antibody (BD Biosciences #610182) and anti-CD44 mouse monoclonal antibody (BD Biosciences #555478), anti-Estrogen receptor alpha rabbit polyclonal antibody (Abcam # ab 75635), anti-ErbB2 mouse monoclonal antibody (Abcam # ab 16901), anti-ErbB2 (phospho Y1248) rabbit polyclonal antibody (Abcam # ab 131104), anti-VEGFA rabbit polyclonal antibody (Abcam # ab183100), anti-STAT5a/b rabbit monoclonal antibody (Abcam # ab 194898), anti-STAT5a (phospho Y694) antibody (Abcam # ab30648). Antibodies used for FACS analysis were FITC mouse anti-human CD24 (BD Biosciences #555427) and APC mouse anti-human CD44 (BD Biosciences #559942). Secondary antibodies used were goat anti-rabbit IgG HRP (Santa- Cruz #sc-2004), rabbit anti-goat IgG-HRP (Santa-Cruz #sc-2922) as well as goat anti-mouse IgG-HRP (Santa-Cruz #sc-2005). Secondary antibodies for confocal immunofluorescence studies were donkey anti- rabbit IgG (H+L) Fluor 546 (Invitrogen), donkey anti-mouse Fluor 488 (Invitrogen), donkey anti-goat IgG-R Rhodamine conjugated (Santa-Cruz #sc-2094) and Alexa Fluor 568 phalloidin (Invitrogen #A12380). The dilutions of antibodies for western blotting analysis are as indicated: 1: 1000 for all primary antibodies. The dilutions for secondary antibodies for western blotting analysis are 1:5000. For immunofluorescence staining: 1:100 for primary antibodies and 1: 200 for secondary antibodies. The dilution for antibodies for FACS analysis is 20:100 as recommended. Other reagents used include: Recombinant human prolactin (rhPRL) (150 ng/ml and 250 ng/ml) used for human cell stimulation was purchased from Feldan Therapeutics (1F-02-008), protein A-Sepharose beads (Amersham Biosciences and GE Healthcare), 12-well plates HTS multi-well insert system format (BD Falcon) and 96-well plates (Corning #3753 and Fisher #7201216). Reagents for lentivirus production and infection of the cells are FastAP (ThermoFisher, cat. No. EF0654), lentiCRISPRv2 (Addgene, cat. No. 52961), pMD2.G (Addgene, cat. No. 12259), Polybrene (hexadimethrine bromide; Sigma-Aldrich, cat. No. 107689-10G), psPAX2 (Addgene, cat. No. 12260). Puromycin dihydrochloride (Thermo Fisher Scientific, cat. No. A1113803). QIAprep Spin Miniprep Kit (QIAGEN, cat. No. 27104). QIAquick Gel Extraction Kit (Qiagen, cat. No. 28704). Quick Ligase (NEB, cat. No. M2200S). T4 ligation buffer (NEB, cat. No. B0202S). T4 PNK (NEB, cat. No. M0201S).

**2. PRLR and non-targeting single-guide sequence annealing and molecular cloning**

LentiCRISPRv2 (Addgene, cat. No. 52961) was digested using Esp3I restriction enzyme (ThermoFisher, cat. No. ER0451), dephosphorylated using FastAP (ThermoFisher, cat. No. EF0654), agarose gel purified and extracted using QIAquick Gel Extraction Kit (QIAGEN, cat. No. 28704). Each single-guide primer sequences below (5’-3’) were phosphorylated using T4 PNK (NEB, cat. No. M0201S), annealed by slow cooling from 65°C to room temperature in T4 ligation buffer (NEB, cat. No. B0202S) and ligated in Esp3I digested lentiCRISPRv2 purified plasmid using Quick Ligase (NEB, cat. No. M2200S). Each sgRNA ligated plasmid was transformed in STBL3 chemically competent *E. coli* (ThermoFisher, cat. No. A10469) and collected from an amplified single bacterial colony using QIAprep Spin Miniprep Kit (QIAGEN, cat. No. 27104) ^60,61^.

| PRLRsg1F | CACCGGTTTGCAGCGAACCTGGACA |
| --- | --- |
| PRLRsg1R | AAACTGTCCAGGTTCGCTGCAAACC |
|  |  |
| PRLRsg2F | CACCGCCATGAATGATACAACCGTG |
| PRLRsg2R | AAACCACGGTTGTATCATTCATGGC |
|  |  |
| PRLRsg3F | CACCGCCACACGGTTGTATCATTCA |
| PRLRsg3R | AAACTGAATGATACAACCGTGTGGC |
|  |  |
| SCR sg1F | CACCGACGGAGGCTAAGCGTCGCAA |
| SCR sg1R | AAACTTGCGACGCTTAGCCTCCGTC |
|  |  |
| SCR sg2F | CACCGCGCTTCCGCGGCCCGTTCAA |
| SCR sg2R | AAACTTGAACGGGCCGCGGAAGCGC |
|  |  |
| SCR sg3F | AAACTTGCGACGCTTAGCCTCCGTC |
| SCR sg3R | AAACCGCCGTTAAGCGGAAACGATC |
|  |  |
|  |  |

**3. Cell culture, cell lines authentication, and Lentiviral infection**

Human breast cancer cells: SKBR3 was obtained from Dr. Morag Park (McGill University). SKBR3, MCF7 cells were maintained in DMEM media (Multicell Invitrogen) containing 10% fetal bovine serum (FBS) (Multicell Invitrogen). For cell line authentication, the genomic DNA (gDNA) was extracted from both MCF-7 and SKBR-3 breast cancer cells following the protocol of Trizol Reagent for DNA isolation from Invitrogen (Cat. # 15596026 & 15596018). The samples were analyzed at the center for Applied Genomics- Genetics Analysis Facility. The results of the analysis were interpreted using ATCC-STR database. The results are attached as excel sheet (Supplementary Materials and Methods). For lentiviral infection: HEK293T cells were transfected with non-targeting sgRNA or PRLR sgRNA and packaging plasmids pMD2.G (Addgene, cat. No. 12259) and psPAX2 (Addgene, cat. No. 12260). After 48hours post-transfection, cell supernatants containing sgRNA lentiviruses were collected. Stable cell lines were then generated via infection with lentiviral sgRNAs in the presence of 8µg/mL polybrene (Sigma Aldrich, cat. No. 107689) and selected for 14 days in presence of 1µg/mL puromycin (Thermo Fisher Scientific, cat. No. A1113803).

**4. Western blotting analysis and Immunoprecipitation**

Total protein lysates were obtained using RIPA lysis buffer (50 mM Tris pH 8, 150 mM sodium chloride, 1% NP-40, 0.5% sodium deoxycholate, 0.1% SDS, 1 mM Na3VO4 and Protease inhibitors cocktail). 30 μg proteins were loaded in the gel. Cell lysates were separated by electrophoresis in 8–12% sodium dodecyl sulphate-polyacrylamide gradient minigel (SDS-PAGE) and electrophoretically transferred to a nitrocellulose membrane. Western blots were probed with the relevant primary antibodies and secondary antibodies. For immunoprecipitation: RIPA lysis buffer was used to obtain the total protein lysates. Mixer of 1 μg of anti-PRLR antibody (D-7), protein A/G beads (20 μl) and 500 μg of cell lysates were incubated for 3h at 4 °C. After washing the beads, western blotting with SDS-PAGE gel was performed with specific antibodies.

**5. Immunofluorescence**

Cells were grown on coverslips with 80% confluency. Fixation process were performed of coverslips coated with cells in 4% Paraformaldehyde for 15 min at room temperature, followed by permeabilization process with 0.1% Triton X-100 (Fisher). Cells were subsequently immune stained with primary antibody for an overnight period at 4 °C and followed by secondary antibody and Dapi for 1 h at room temperature. Mounting media (Lerner # 13800) was used to mount the coverslips and stored at 4 °C. Confocal microscopy was performed using Zeiss LSM 780 confocal microscope equipped with a Plan- Apochromat x63- 1.4 oil immersion objective.

**6. Immunohistochemistry and scoring**

Immunohistochemical staining was performed on paraffin embedded slides. After deparaffinization and rehydration slides were immersed in retrieval solution (sodium citrate 10 mM, pH 6.0 buffer). The slides were incubated in hydrogen peroxide blocks, followed by Ultra V Block. Slides were incubated with an indicated antibody. Ultra-Vision LP Detection System HRP Polymer & DAP Plus Chromogen (Thermo Fisher Scientific, Fremont CA) was used for detection. The slides were scanned using Aperio XT slide scanner (Leica Biosystems). Quantitative IHC scoring systems were used to evaluate the expression of different markers. In brief, a representative annotated malignant region was selected for each core using images of 40 × magnification from digital IHC-stained slides. The mean positive pixel count (PPC) for each representative region was obtained using positive pixel count (PPC) algorithm (Aperio). The percentage of positive stained cells was calculated in four different regions of the slide and the mean average was plotted using graph pad prism. The immunostaining score for PRLR expression was done as previously described^30^ . The immunostaining score for Ki-67 expression was done as previously described^62^. The immunostaining score for expression the markers (ER, HER2, E-cad, CK18, CK5/6, Vim, CD44 and VEGFA) was done as previously described ^63,64^.

**7. ALDEFLOUR assay**

According to the manufacturer protocol, the ALDEFLUOR kit (Cat#01700, Stemcell Technologies) was used to measure ALDH activity. In brief, 1 × 106/ml cells were re-suspended in ALDH assay buffer. This is followed by adding activated 5μl ALDH substrate (Bodipy-Aminoacetaldehyde) to each sample and kept for incubation for 45 min at 37 °C. For negative control tubes, 5 μl of a 1.5mM Diethylamino benzaldehyde (DEAB) was added to samples containing ALDH substrate. The gates were established based on this negative control for each specific cell line. This negative control gives the baseline fluorescence of these cells. Accuri C6 flow cytometer ma- chine was used to detect ALDH positive cells and data analyzed using Flowjo software.

**8. Flow cytometry analysis**

Adherent cells were dissociated into single cells by trypsin-EDTA and filtered through a 40μm nylon mesh (BD Biosciences, San Diego, CA, USA). 1 × 106 cells were washed with PBS containing 0.5% FBS, incubated with APC mouse anti-human CD44 (BD Biosciences #559942) and FITC mouse anti-human CD24 (BD Biosciences #555427). Cells were then washed with 0.5%PBS-FBS for three times. After washing, cells were analyzed with Accuri C6 flow cytometer (BD Biosciences) and Flowjo software (Tree Star Inc). The proliferation platform of this software draws gates in order to separate each generation based on unstained control.

**9. Sulforhodamine B assays**

Cells cultured in 96-well plates and treated or left untreated with either tamoxifen (0-200) μM or lapatinib (0-25μM). Tamoxifen citrate salt was purchased from Sigma-Aldrich (Prod. No. T9262), Lapatinib was purchased from Sigma-Aldrich (Prod. No. CDS022971-25MG). The preparation of each drug was performed following the company’s instructions. After completion of the treatment period the cells were fixed with 10% tricholoroacetic acid in complete media and stained for 30 minutes with 0.4% (wt/vol) SRB dissolved in 1% acetic acid. Unbound dye was removed by four washes with 1% acetic acid, and protein-bound dye was extracted with 10 mM unbuffered Tris base for determination of absorbance at 490 nm using a spectrophotometer according to the protocol.

**10. Animal models**

All experimental animal work was performed in a specific- pathogen-free animal facility according to the guidelines and ethical regulations of the Research Institute McGill University Health Centre approved animal used protocol (#2014-7492) in accordance with Canadian Council of animal care guidelines.

**11. Mammary fat pad NOD-SCID mouse xenografts**

Twenty-four female NOD-SCID mice were purchased from Charles River Laboratories (Saint-Constant, QC, Canada), housed and maintained under specific pathogen-free conditions (RI-MUHC animal facility). The mice were randomly divided into two groups (n = 6 mice per group) and procedures were conducted unblinded. At 7 to 9 weeks of age, the first group was injected in the fourth-right mammary fat pad with 1 × 106 NT (MCF-7/NT or SKBR-3/NT) cell lines and the second group was injected with (MCF-7/PRLRKO (SG1) or SKBR-3/PRLRKO (SG3) cell lines. No estrogen supplementation was used for the MCF-7 model ^41^. Tumor growth was monitored up to 48 days (MCF-7 model) and up to 21 days (SKBR-3 model) after cell transplantation. When tumors were detectable, tumor size was measured with a Vernier caliper (Mitutoyo, Kawasaki, Japan) and calculated using the formula [length + width^2]/2. Mice were sacrificed by CO2 asphyxiation.

**12. MCF-7 and SKBR-3 tail vein NSG mouse xenografts**

Eighteen Female NSG mice were purchased from Charles River Laboratories (Saint-Constant, QC, Canada), housed and maintained under specific pathogen-free conditions (RI-MUHC animal facility). The mice were randomly divided into two groups (n = 9 mice per group) and procedures were conducted unblinded. At 7 to 9 weeks of age, the first group was injected in the tail vein with 1 ×106 MCF-7/NT and the second group were injected with 1 × 106 MCF-7/PRLRKO (SG1). Mice were monitored up to 4 weeks after injection. For SKBR-3 model ten Female NSG mice were purchased from Charles River Laboratories. The mice were randomly divided into two groups (n = 5 mice per group) and procedures were conducted unblinded. At 7 to 9 weeks of age, the first group was injected in the tail vein with 1 ×106 SKBR-3/NT and the second group were injected with 1 × 106 SKBR-3/PRLRKO (SG3). Mice were monitored up to 5 weeks after injection Mice were sacrificed by CO2 asphyxiation and lungs and other organs were collected.
